# Integrative multi-environmental genomic prediction in apple

**DOI:** 10.1093/hr/uhae319

**Published:** 2024-11-20

**Authors:** Michaela Jung, Carles Quesada-Traver, Morgane Roth, Maria José Aranzana, Hélène Muranty, Marijn Rymenants, Walter Guerra, Elias Holzknecht, Nicole Pradas, Lidia Lozano, Frédérique Didelot, François Laurens, Steven Yates, Bruno Studer, Giovanni A L Broggini, Andrea Patocchi

**Affiliations:** Fruit Breeding, Agroscope, Mueller-Thurgau-Strasse 29, 8820 Waedenswil, Switzerland; Molecular Plant Breeding, Institute of Agricultural Sciences, ETH Zurich, Universitaetstrasse 2, 8092 Zurich, Switzerland; Molecular Plant Breeding, Institute of Agricultural Sciences, ETH Zurich, Universitaetstrasse 2, 8092 Zurich, Switzerland; INRAE, Research Unit for Genetics and Improvement of Fruit and Vegetable (GAFL), 67 Allée des Chênes, 84143 Montfavet, France; Centre for Research in Agricultural Genomics (CRAG) CSIC-IRTA-UAB-UB, Campus UAB, Bellaterra, 08193 Barcelona, Spain; IRTA (Institut de Recerca i Tecnologia Agroalimentàries), Caldes de Montbui, 08140 Barcelona, Spain; Univ Angers, Institut Agro, INRAE, IRHS, SFR QuaSaV, F-49000 Angers, France; Better3fruit N.V., Steenberg 36, 3202 Rillaar, Belgium; Laboratory for Plant Genetics and Crop Improvement, Division of Crop Biotechnics, Department of Biosystems, University of Leuven, Willem de Croylaan 42 - bus 2427, 3001 Leuven, Belgium; Research Centre Laimburg, Institute for Fruit Growing and Viticulture, Laimburg 1, 39040 Auer, Italy; Research Centre Laimburg, Institute for Fruit Growing and Viticulture, Laimburg 1, 39040 Auer, Italy; Centre for Research in Agricultural Genomics (CRAG) CSIC-IRTA-UAB-UB, Campus UAB, Bellaterra, 08193 Barcelona, Spain; IRTA (Institut de Recerca i Tecnologia Agroalimentàries), Caldes de Montbui, 08140 Barcelona, Spain; Unité expérimentale Horticole, INRAE, F-49000 Angers, France; Univ Angers, Institut Agro, INRAE, IRHS, SFR QuaSaV, F-49000 Angers, France; Molecular Plant Breeding, Institute of Agricultural Sciences, ETH Zurich, Universitaetstrasse 2, 8092 Zurich, Switzerland; Molecular Plant Breeding, Institute of Agricultural Sciences, ETH Zurich, Universitaetstrasse 2, 8092 Zurich, Switzerland; Molecular Plant Breeding, Institute of Agricultural Sciences, ETH Zurich, Universitaetstrasse 2, 8092 Zurich, Switzerland; Fruit Breeding, Agroscope, Mueller-Thurgau-Strasse 29, 8820 Waedenswil, Switzerland

## Abstract

Genomic prediction for multiple environments can aid the selection of genotypes suited to specific soil and climate conditions. Methodological advances allow effective integration of phenotypic, genomic (additive, nonadditive), and large-scale environmental (enviromic) data into multi-environmental genomic prediction models. These models can also account for genotype-by-environment interaction, utilize alternative relationship matrices (kernels), or substitute statistical approaches with deep learning. However, the application of multi-environmental genomic prediction in apple remained limited, likely due to the challenge of building multi-environmental datasets and structurally complex models. Here, we applied efficient statistical and deep learning models for multi-environmental genomic prediction of eleven apple traits with contrasting genetic architectures by integrating genomic- and enviromic-based model components. Incorporating genotype-by-environment interaction effects into statistical models improved predictive ability by up to 0.08 for nine traits compared to the benchmark model. This outcome, based on Gaussian and Deep kernels, shows these alternatives can effectively substitute the standard genomic best linear unbiased predictor (G-BLUP). Including nonadditive and enviromic-based effects resulted in a predictive ability very similar to the benchmark model. The deep learning approach achieved the highest predictive ability for three traits with oligogenic genetic architectures, outperforming the benchmark by up to 0.10. Our results demonstrate that the tested statistical models capture genotype-by-environment interactions particularly well, and the deep learning models efficiently integrate data from diverse sources. This study will foster the adoption of multi-environmental genomic prediction to select apple cultivars adapted to diverse environmental conditions, providing an opportunity to address climate change impacts.

## Introduction

Since the introduction of genomic selection [1], the genome-wide selection based on thousands of markers has resulted in increased genetic gain, and this approach is progressively becoming an integral component of modern crop breeding programs [2, 3]. To predict the genomic estimated breeding values for genomic selection, marker effects are frequently estimated using the well-established genomic best linear unbiased predictor (G-BLUP) approach [4]. For genomic prediction across environments, increased predictive ability has been demonstrated by utilizing G-BLUP to incorporate the main marker effects and interaction effects of markers and environments [5, 6]. The interaction between markers and environments provides a mathematical representation of the natural phenomenon of genotype-by-environment interaction, which results from the variability in the genotype performance ranking across different environmental conditions. Despite numerous reports of successful phenotypic performance prediction using molecular markers in perennial crops such as apple [7–10], genotype-by-environment interaction has been often overlooked in genomic prediction of apple traits.

The most comprehensive study conducted thus far to investigate the influence of genotype-by-environment interaction on genomic predictive ability in apple, conducted by Jung *et al.* [11], was achieved by the establishment of the apple reference population, known as the apple REFPOP [12]. Across the numerous phenotypic traits assessed in the apple REFPOP, genotype-by-environment interaction explained up to 24% of the phenotypic variance, and the incorporation of genotype-by-environment interaction into G-BLUP resulted in a predictive ability increase of up to 0.07 [11]. The challenge of building multi-environmental datasets, coupled with the computational costs tied to the structural complexity of genomic prediction models accommodating genotype-by-environment interaction, has likely limited the use of such models in practice.

Recent software advances that reduce computational time could enable broader adoption of multi-environmental genomic prediction models in plant breeding (Costa-Neto, Fritsche-Neto, *et al.*, 2021; [13]). Empirical comparisons between the well-established R package ‘BGLR’ [14] and the newer R package ‘BGGE’ [13], both of which apply the same model structures based on G-BLUP, revealed comparable predictive abilities, but ‘BGGE’ was up to five times faster [13]. In addition to G-BLUP, covariance matrices, alternatively referred to as relationship matrices or kernels, can be estimated using approaches that capture nonlinearity in the relationships between phenotype and genotype. The nonlinear Gaussian kernel and the Deep kernel (also known as the arc-cosine kernel) have demonstrated superior performance compared to G-BLUP, showing reduced computational time and increased predictive ability in maize and wheat datasets (Costa-Neto, Fritsche-Neto, *et al.*, 2021; [15]).

In addition to the commonly used genomic effects of molecular markers, the advancements in software have introduced straightforward options for incorporating additional sources of variation into genomic prediction models (Costa-Neto, Fritsche-Neto, *et al.*, 2021; Costa-Neto, Galli, *et al.*, 2021). Using the natural and orthogonal interactions (NOIA) approach, marker values can be split into additive values and dominance deviations that allow for orthogonal partition of variances, which implies that the proportions of additive genomic effects remain constant even when dominance effects are incorporated into the genomic prediction model [16]. The incorporation of dominance effects into genomic prediction models is typically done by the use of relationship matrices, as proposed by Vitezica *et al.* [17, 18]. Unlike other approaches to construct relationship matrices for dominance (e.g. [18]), the NOIA approach does not assume Hardy–Weinberg equilibrium, which makes it particularly suitable for populations such as those resulting from crosses [17]. In apple, the inclusion of nonorthogonal dominance effects under the assumption of Hardy–Weinberg equilibrium did not affect predictive ability [19]. However, combining dominance effects applying the NOIA approach along with a fixed effect of inbreeding has demonstrated improved genomic predictive ability in maize and sugarcane [20, 21]. Additionally, incorporating nongenetic effects derived from large-scale assessment of environmental attributes (i.e. envirotyping, resulting in environmental covariates also called enviromic markers [22, 23]) into genomic prediction models can improve the estimation of similarities between environments and genotype-by-environment interaction. This enhancement not only leads to increased predictive ability, but also offers a more comprehensive understanding of the complex interplay between genetic and environmental factors (Costa-Neto, Fritsche-Neto, *et al.*, 2021; [5]). The enviromic-based effects, as well as the marker-based effects expressed as standard genomic, orthogonal additive, and dominance effects, can all be studied as extensions of G-BLUP using conventional statistical genomic prediction model frameworks, which simplifies their integration into the modeling process.

Deep learning approaches have emerged as an alternative to conventional statistical genomic prediction models. The literature review of Montesinos-López *et al.* [24] on the application of deep learning for genomic selection showed no distinct superiority of deep learning approaches in terms of predictive ability compared to conventional genomic prediction models, unless very large datasets were used. However, deep learning models allow for effective integration of data from diverse sources, but they can also become impractical for datasets containing many variables, leading to computational complexity and overfitting. In plant breeding, datasets comprising thousands of markers are compiled, and dimensional reduction may help simplify marker information for deep learning [25]. In the study by Jurado-Ruiz *et al.* [26], the use of a small subset of associated markers was critical for accurate predictions of apple shape when deploying neural networks. The potential application of deep learning for multi-environmental genomic prediction of diverse quantitative apple traits has yet to be examined.

This study aims to conduct a comprehensive comparison between conventional statistical models that integrate genomic- and enviromic-based effects and a deep learning approach for multi-environmental genomic prediction of apple traits. The subjects of prediction were eleven quantitative traits related to phenology, productivity, and fruit quality, which were measured from the apple REFPOP during five years at up to five locations, i.e. up to 25 environments (defined as combinations of location and year). The increased extent of the apple REFPOP dataset across environments allows an evaluation of different modeling techniques to harness the full potential of these data for accurate prediction of phenotypic traits. The main objectives of the study were: (i) to evaluate the relative contribution of different model components, i.e. random effects and feature streams, for the statistical and deep learning genomic prediction models, and (ii) to assess and compare predictive abilities of these models. By addressing these two crucial factors, this research aims to provide insights into the strengths and limitations of statistical models and deep learning to identify the best modeling solutions for the selection of apple cultivars adapted to diverse environmental conditions.

## Results

### Dataset composition

**Figure 1:**
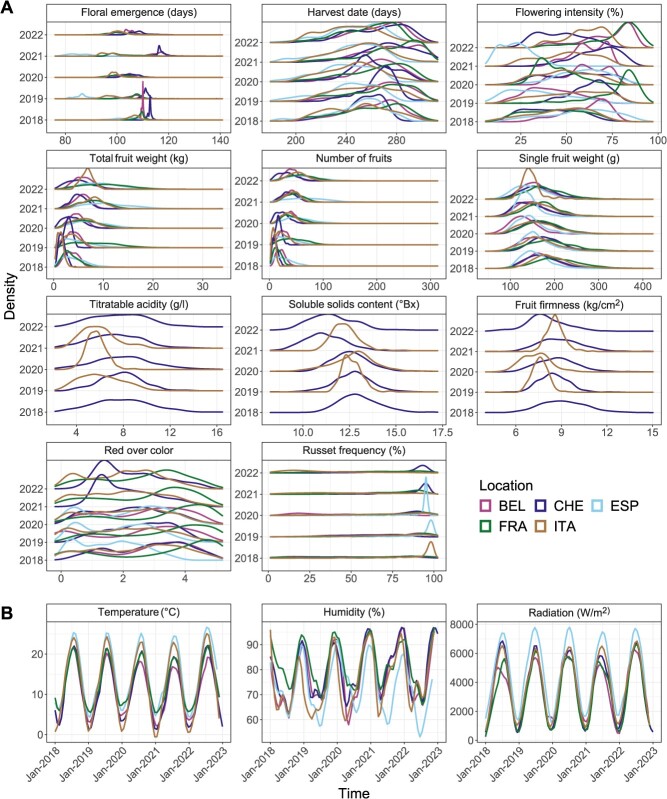
Phenotypic and weather data distributions. **A,** Density estimates for the adjusted means of eleven phenotypic traits from five locations and five years of measurement. The locations correspond to Belgium (BEL), Switzerland (CHE), Spain (ESP), France (FRA), and Italy (ITA). **B,** Local regression curves spanning five years estimated from daily temperature means, daily humidity means, and daily radiation sums. Colors correspond to legend in A.

From the eleven phenotypic traits assessed in the apple REFPOP over five years and at a maximum of five locations, two environment–trait combinations were excluded due to very low values of the environment-specific clonal mean heritability (${H}^2<0.1$). The excluded combinations included phenotypic measurements for floral emergence in Spain in 2020 (${H}^2=0.036$) and flowering intensity in France in 2021 (${H}^2=0.002$). Consequently, phenotypic estimates were generated from a minimum of eight environments for titratable acidity, soluble solids content, and fruit firmness, while harvest date, total fruit weight, number of fruits, and single-fruit weight were evaluated across the maximum number of environments, totaling 25 (Table S1). Various shapes of distributions and consistent patterns of Pearson’s correlations were observed for the adjusted means of phenotypic traits over years and locations (Fig. 1a, Fig. S1, Fig. S2).

For the weather variables, moderate differences were observed in daily temperature means, daily humidity means, and daily radiation sums between years and locations (Fig. 1b). Consequently, these data were summarized based on phenology, meaning the data was split into two periods: the first 80 days until 90% of the genotypes flowered, and the following days until 90% of the genotypes were harvested (Fig. S3). After preprocessing the soil variables, the final enviromic dataset included 28 environmental covariates for weather and soil.

### Relationship matrices

**Figure 2:**
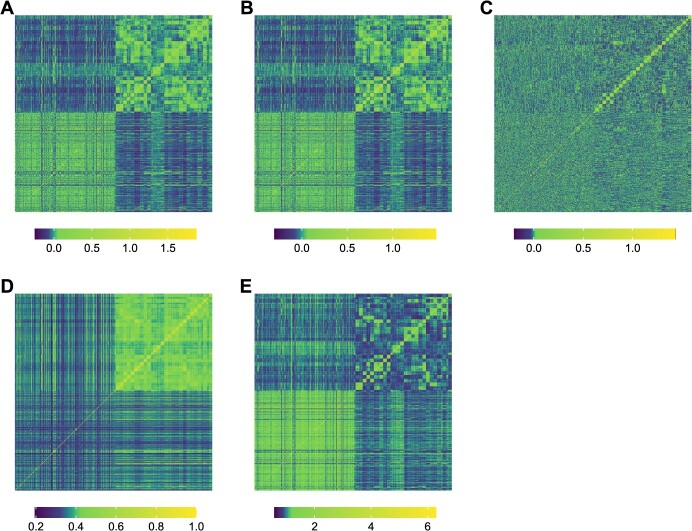
Heat maps of the genomic relationship matrices. **A,** Standard genomic relationship matrix ${\boldsymbol{K}}_{\boldsymbol{G}}$ based on a marker matrix using the standard coding for biallelic SNPs (allele dosage values of 0, 1, and 2). **B,** Additive genomic relationship matrix ${\boldsymbol{K}}_{\boldsymbol{A}}$ based on marker matrix using the additive coefficients. **C,** Dominance genomic relationship matrix ${\boldsymbol{K}}_{\boldsymbol{D}}$ based on marker matrix using the dominance coefficients. The matrices in A–C were constructed using the G-BLUP approach. **D,** Standard genomic relationship matrix ${\boldsymbol{K}}_{{\boldsymbol{G}}_{\boldsymbol{G}\boldsymbol{K}}}$ constructed deploying the Gaussian kernel (GK). **E,** Standard genomic relationship matrix ${\boldsymbol{K}}_{{\boldsymbol{G}}_{\boldsymbol{DK}}}$ based on the Deep kernel (DK). The lower-left and upper-right quadrants show the apple REFPOP accessions and progenies, respectively.

**Figure 3:**
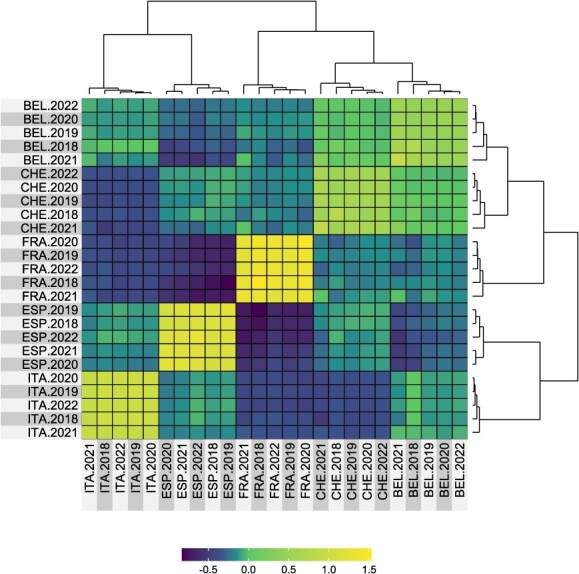
The enviromic relationship matrix ${\boldsymbol{K}}_{\boldsymbol{W}}$ constructed from the environmental covariates for weather and soil using G-BLUP. Environments (combinations of location and year) were grouped applying hierarchical clustering.

Implementation of the G-BLUP approach resulted in the standard genomic relationship matrix ${\boldsymbol{K}}_{\boldsymbol{G}}$ (based on standard allele coding with allele dosage values of 0, 1, and 2), the additive genomic relationship matrix ${\boldsymbol{K}}_{\boldsymbol{A}}$, and the dominance genomic relationship matrix ${\boldsymbol{K}}_{\boldsymbol{D}}$ (Fig. 2a, b, and c). The heat maps of these matrices depicted a strong similarity between ${\boldsymbol{K}}_{\boldsymbol{G}}$ and ${\boldsymbol{K}}_{\boldsymbol{A}}$ (Fig. 2a and b). The lower-left quadrant of matrices ${\boldsymbol{K}}_{\boldsymbol{G}}$ and ${\boldsymbol{K}}_{\boldsymbol{A}}$ comprised the apple REFPOP accessions, revealing only subtle differences between these genotypes. The upper-right quadrant of matrices ${\boldsymbol{K}}_{\boldsymbol{G}}$ and ${\boldsymbol{K}}_{\boldsymbol{A}}$ visualized the apple REFPOP progenies grouped according to their biparental origin. The progeny groups were evident in the matrix ${\boldsymbol{K}}_{\boldsymbol{D}}$, but no further strong relationships between genotypes were visually observed. ${\boldsymbol{K}}_{\boldsymbol{A}}$ and ${\boldsymbol{K}}_{\boldsymbol{D}}$ showed the mean of their matrix values close to zero and the mean of the diagonal of 1. Gaussian kernel and Deep kernel, used as alternative approaches to G-BLUP, resulted in matrices ${\boldsymbol{K}}_{{\boldsymbol{G}}_{\boldsymbol{G}\boldsymbol{K}}}$ and ${\boldsymbol{K}}_{{\boldsymbol{G}}_{\boldsymbol{DK}}}$ (Fig. 2d and e) that were visually similar to the ${\boldsymbol{K}}_{\boldsymbol{G}}$ and ${\boldsymbol{K}}_{\boldsymbol{A}}$ matrices implemented using G-BLUP (Fig. 2a and b), although some differences were observed particularly for the Gaussian kernel approach (Fig. 2d). Application of the G-BLUP to the enviromic dataset of 28 environmental covariates resulted in the enviromic relationship matrix ${\boldsymbol{K}}_{\boldsymbol{W}}$ (Fig. 3). Hierarchical clustering of the matrix ${\boldsymbol{K}}_{\boldsymbol{W}}$ showed five clusters of environments, each cluster referring to one of the orchard locations.

### Contribution of the model components

**Figure 4:**
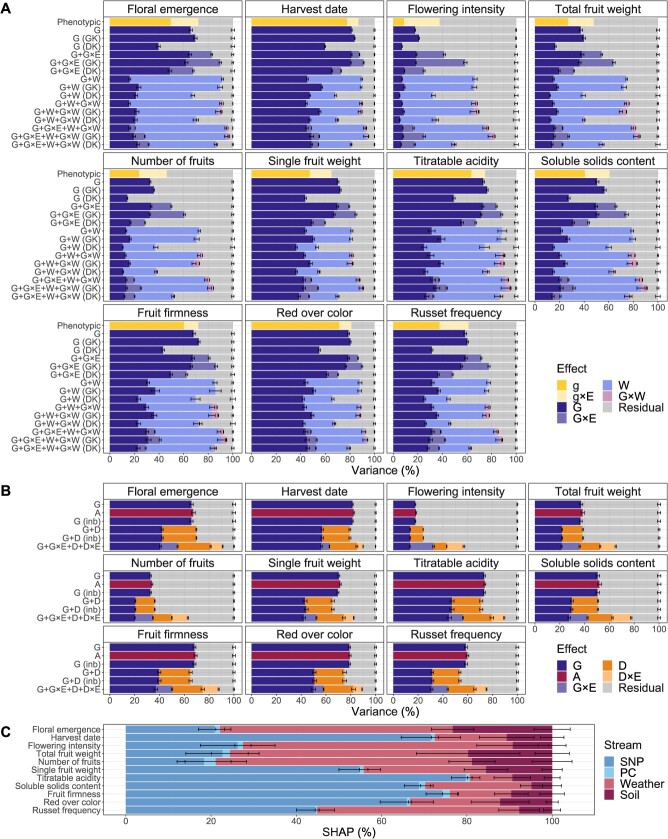
Relative contribution of different model components estimated for eleven traits. **A,** Average proportions of phenotypic variance related to genotypic (g) and genomic (G) effects, their interactions (×) with the vector of environments (E), the enviromic effects (W), the interaction effects G × W, as well as the residual effect extracted from the statistical genomic prediction model fits. The relationship matrices for the different effects in the statistical genomic prediction models were constructed using the G-BLUP approach or, where indicated, the Gaussian kernel (GK) or Deep kernel (DK). The statistical genomic prediction models were compared with a model based on phenotypic data (Phenotypic). Error bars correspond to standard deviation around the mean. **B,** Average proportions of phenotypic variance related to genomic (G), additive (A), and dominance (D) effects, their interactions (×) with the vector of environments (E), and the residual effect extracted from the statistical genomic prediction model fits. The model structures G and G + D were additionally extended with the fixed effect of inbreeding (inb). The relationship matrices for the different effects were based on G-BLUP. Error bars correspond to standard deviation around the mean. The results for the benchmark model G are the same as shown in A. **C** Relative contribution of the SNP, PC, weather, and soil feature streams estimated using SHAP for the deep learning genomic prediction model. Error bars correspond to standard deviation around the mean.

Decomposition of the phenotypic variance using linear mixed models by incorporating random effects for the vector of genotypes (i.e. genotypic effects) and genotype-by-environment interaction revealed that the proportion of phenotypic variance explained by the genotypic effects ranged from 9% for flowering intensity to 78% for harvest date (Fig. 4a, Table S2). In contrast, the largest proportion of phenotypic variance explained by genotype-by-environment interaction was observed for flowering intensity (29%). The lowest proportion of genotype-by-environment interaction variance (9%) was found for harvest date. The total variance explained by both genotypic and genotype-by-environment interaction effects reached 64% on average across traits (Table S3).

For the statistical genomic prediction based on G-BLUP, linear mixed model structures resulted from the application of the relationship matrices ${\boldsymbol{K}}_{\boldsymbol{G}}$, ${\boldsymbol{K}}_{\boldsymbol{A}}$, and ${\boldsymbol{K}}_{\boldsymbol{D}}$, representing genomic (G), additive (A), and dominance (D) effects, respectively. Various proportions of phenotypic variance related to these random effects and their interactions (×) were extracted from the model fits (Fig. 4b, Table S2). Due to its model structure, the simplest genomic prediction model (used as a benchmark) was labeled as G, and its random genomic effects accounted for an average of 58% of the variance across traits (Table S3). Across all traits, model A explained ~1% more variance compared to model G (Table S3). Including the fixed effect of inbreeding in model G, leading to model G (inb), resulted in the same proportion of explained variance of 58% as for model G (Table S3). For the models G + D and G + D (inb), the average total proportion of variance explained by the model components G and D across traits was 1% lower than that of model G (Table S3). The model G + G × E + D + D × E, on average across traits, explained a proportion of variance 21% greater than that explained by model G (Table S3).

The model G + G × E based on G-BLUP, including interactions with the environment, explained, on average across traits, a proportion of variance 14% greater than that explained by model G (Table S3). Specifically, the effect G accounted for variance ranging from 19% for flowering intensity to 81% for harvest date, and G × E explained variance ranging from 6% for harvest date to 23% for flowering intensity (Fig. 4a, Table S2).

The enviromic effects (W) and the interaction effects G × W were implemented applying the relationship matrix ${\boldsymbol{K}}_{\boldsymbol{W}}$ based on G-BLUP in the model structures G + W, G + W + G × W, and G + G × E + W + G × W, and these models explained, on average across traits, 24%, 25%, and 30% more variance than model G, respectively (Fig. 4a, Table S3). For the most complex model G + G × E + W + G × W, the proportions of variance explained by the interaction effects G × E and G × W were modest, ranging from 4% to 9% for G × E and 2%–4% for G × W (Table S2).

**Figure 5:**
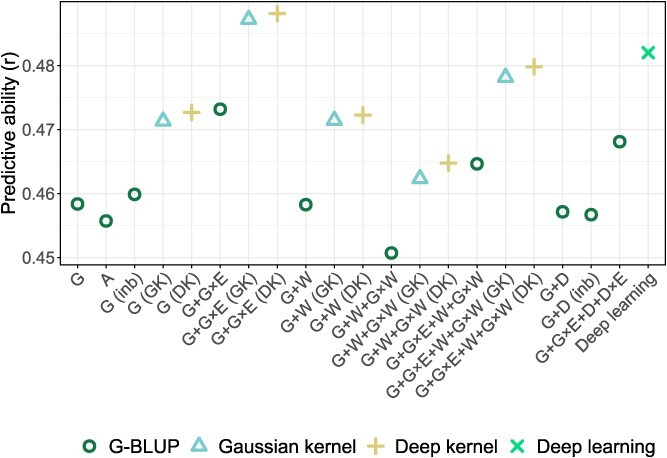
Comparison of predictive ability averaged across all studied traits. The statistical genomic prediction models were based on combinations of the genomic (G), additive (A), dominance (D), and enviromic (W) effects, interactions (×) with the vector of environments (E), and interactions between the genomic and enviromic effects (G × W). The model structures G and G + D were additionally extended with the fixed effect of inbreeding (inb). The relationship matrices for the different effects in the statistical genomic prediction models were constructed using the G-BLUP approach or, where indicated, the Gaussian kernel (GK) or Deep kernel (DK). The y-axis was truncated to provide a detailed model comparison. See Table S6 for a comparison of the predictive ability for each trait.

When comparing models based on G-BLUP with their counterparts implementing Gaussian kernel using the relationship matrix ${\boldsymbol{K}}_{{\boldsymbol{G}}_{\boldsymbol{G}\boldsymbol{K}}}$ (model structures labeled with GK), the models G (GK), G + G × E (GK), and G + G × E + W + G × W (GK) demonstrated an average increase in explained variance of 3%, 7%, and 3% across traits, respectively (Fig. 4a, Table S3). However, the models G + W (GK) and G + W + G × W (GK) resulted in an average decrease in explained variance of up to 1% (Table S3). On average over traits, the model structures based on Deep kernel implementing the relationship matrix ${\boldsymbol{K}}_{{\boldsymbol{G}}_{\boldsymbol{DK}}}$ (model structures labeled with DK) exhibited a strong decrease in the proportion of variance explained by the genomic- and enviromic-based random effects when compared to their counterparts utilizing G-BLUP, namely −22% for G (DK), −19% for G + G × E (DK), −26% for G + W (DK), −25% for G + W + G × W (DK), and −17% for G + G × E + W + G × W (Table S3).

The applied deep learning genomic prediction model integrated marker and enviromic data through four feature streams, namely single nucleotide polymorphism (SNP), principal component (PC), weather, and soil streams, and the estimation of Shapley additive explanations (SHAP) revealed the relative mean importance of these feature streams (Fig. 4c, Table S4). Across all traits, the relative SHAP contributions were 50% for the SNP stream, 1% for the PC stream, 36% for the weather stream, and 13% for the soil stream. The relative SHAP contribution for the SNP stream ranged from 18% to 26% for floral emergence and the productivity traits (flowering intensity, total fruit weight, and number of fruits) to 80% for titratable acidity. For the PC stream, the relative SHAP contribution ranged between 0% for russet frequency and 3% for number of fruits. The lowest weather stream contribution of 10% was found for titratable acidity, while the largest contribution of the weather stream of 55%–63% was found for floral emergence and the productivity traits (flowering intensity, total fruit weight, and number of fruits). The relative SHAP contribution for the soil stream ranged between 5% for soluble solids content and 19%–23% for floral emergence and two productivity traits (total fruit weight and number of fruits). An abundance of SNPs displaying high absolute mean SHAP were found for harvest date, titratable acidity, and red over color (Fig. S4, Fig. S5). For harvest date, three SNPs with the highest absolute mean SHAP of 0.002 were located on chromosome 3 at 29.2 Mb (AX-115250472), 30.7 Mb (AX-115366114), and 30.8 Mb (AX-115233388). The three SNPs with the highest absolute mean SHAP of 0.003 for titratable acidity were found on chromosome 8 at 10.7 Mb (AX-115276534), 10.8 Mb (AX-115254093), and 11.8 Mb (AX-115519462). For red over color, the three SNPs with the highest absolute mean SHAP of 0.005 were located on chromosome 9 at 33.8 Mb (AX-105213720, AX-115558498) and 35.6 Mb (AX-115370846).

### Predictive ability

Assessment of genomic prediction model performance using 5-fold cross-validation showed that the average predictive ability across traits ranged from 0.45 to 0.49 for the compared models (Fig. 5, Table S5). Based on these average predictive abilities, the model G + W + G × W emerged as the least efficient, with an average predictive ability across traits of 0.45. Models A, G (inb), G + W, G + W + G × W (GK), G + W + G × W (DK), G + G × E + W + G × W, G + D, and G + D (inb) demonstrated equivalent average predictive ability across traits, with a value of 0.46, comparable to the benchmark model G based on G-BLUP. Average predictive ability across traits of 0.47 was found for the models G (GK), G (DK), G + G × E, G + W (GK), G + W (DK), and G + G × E + D + D × E. The models G + G × E + W + G × W (GK), G + G × E + W + G × W (DK), and deep learning provided additional improvement with the average predictive ability across traits of 0.48. The models G + G × E (GK) and G + G × E (DK) showed the highest average predictive ability across traits of 0.49.

**Figure 6:**
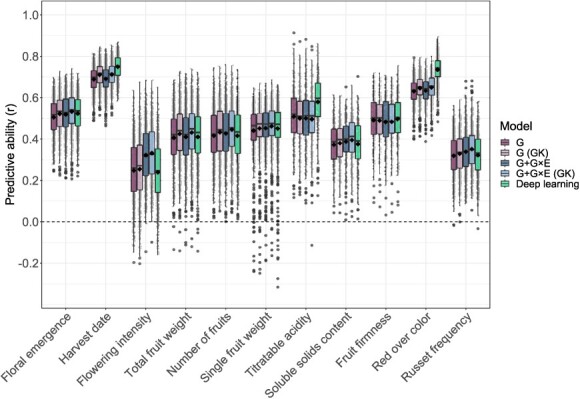
Boxplots of predictive abilities for eleven traits estimated using statistical and deep learning genomic prediction models. The statistical genomic prediction models were based on combinations of the genomic effects (G) and their interactions with the vector of environments (G × E). The relationship matrices for the different effects in the statistical genomic prediction models were constructed using the G-BLUP approach or, where indicated, the Gaussian kernel (GK). Twenty-five predictive ability estimates were generated for each available environment (up to 625 estimates per trait), and their average was displayed as black diamonds for each model and trait. Jittered points (gray) show all predictive ability estimates for each trait.

For four models selected for an in-depth comparison with the benchmark model G based on their performance and characteristics (G (GK), G + G × E, G + G × E (GK), and deep learning), strong differences in average predictive ability were observed among the examined traits (Fig. 6, Table S6). Flowering intensity and russet frequency were at the lower end of the predictive ability spectrum, while harvest date and red over color were at the upper end. Compared to model G, the model G (GK) showed an increase in average predictive ability of 0.01–0.02 for most traits, but no improvement in predictive ability was found using this model for titratable acidity and fruit firmness. Model G + G × E led to an increase in average predictive ability of 0.07 for flowering intensity and 0.01–0.02 for floral emergence, number of fruits, single-fruit weight, soluble solids content, and russet frequency. It showed no improvement for harvest date, total fruit weight, titratable acidity, fruit firmness, and red over color. Model G + G × E (GK) demonstrated an additional improvement in average predictive ability of 0.01–0.02 compared to model G + G × E for all traits, except for titratable acidity and fruit firmness. For these two traits, the incorporation of the G × E effect led to a decrease in average predictive ability by 0.01 in both tested models, G + G × E and G + G × E (GK), compared to model G. The deep learning genomic prediction model demonstrated higher predictive abilities than model G for five out of the eleven traits studied. For harvest date, titratable acidity and red over color, the deep learning genomic prediction model outperformed all statistical genomic prediction models tested. The increase in average predictive ability compared to model G was 0.06 for harvest date, 0.07 for titratable acidity, and 0.10 for red over color.

## Discussion

This study provides insights into the complexities of multi-environmental genomic prediction in quantitative apple traits. The incorporation of different sources of variation in the form of model components, and the comparison of predictive abilities between statistical genomic prediction models and a deep learning approach contribute to advancing the understanding of efficient genomic prediction methodologies. The findings highlight the need for a nuanced approach, considering the specific traits and modeling approaches in plant breeding applications.

### Modeling genotype-by-environment interaction

In the context of genomic prediction across environments (defined as combinations of location and year), this work underscored a detectable improvement in predictive ability when employing genomic prediction models based on G-BLUP that integrate both main marker effects and the interaction effects of markers and environments, as it has been described by previous studies [5, 6, 11]. Compared to the benchmark genomic prediction model implementing exclusively the main marker effects, Jung *et al.* [11] reported up to 0.07 increase in predictive ability for apple traits by integrating the random effects for G × E using the software package ‘BGLR’ [14]. In this study deploying the newer software ‘BGGE’ [13], an analogous model comparison based on the same plant material but including two additional years of phenotypic data, showed comparable improvements in predictive ability of up to 0.07. Average predictive ability across eleven studied traits for models incorporating G × E using G-BLUP was 0.01 lower compared to the average predictive ability for the same traits reported previously (Table S6, [11]). As the predictive ability of G × E models based on G-BLUP was similar in ‘BGLR’ and ‘BGGE’ [13], the difference in predictive ability was likely due to the changes in the phenotypic dataset between the compared studies.

The inclusion of G × E effects led to an increase in predictive ability, which was associated with a higher proportion of variance explained by the random effects (Fig. 4). However, the improvement in predictive ability was disproportionately smaller compared to the increase in explained variance. This discrepancy between the substantial rise in explained variance and the modest gain in predictive ability was observed across all the statistical genomic prediction models studied, contrary to expectations (Costa-Neto, Fritsche-Neto, *et al.*, 2021). It might be explained by the fact that variance was estimated using the training sets, while predictive ability was evaluated on the validation sets. This suggests that the model, although effectively capturing patterns in the training set, did not generalize well to the validation set, resulting in limited improvements in predictive ability for the validation set.

### Dominance effects

Previous study by Kumar *et al.* [19] showed similar predictive ability between genomic prediction models with and without dominance effects when analyzing quantitative traits in apple. In their work, dominance effects were modeled using nonorthogonal coefficients and under the assumption of Hardy–Weinberg equilibrium. In contrast, our study implemented orthogonal dominance coefficients that do not assume Hardy–Weinberg equilibrium, leading to the expectation of improved predictive ability [20, 21]. However, despite this implementation, only limited improvement in predictive ability was observed for the G + D and G + G × E + D + D × E models, as well as for models incorporating inbreeding (Table S5).

Orthogonal partitioning of variances implies that the proportions of additive genomic effects remain unchanged when additional effects, such as dominance, are introduced into the genomic prediction model [16]. Despite using the NOIA procedure for orthogonal partitioning of additive and dominant variances that do not assume Hardy–Weinberg equilibrium [16, 17], our results indicate nonorthogonality when comparing models G, A, and G + D. Specifically, the comparison of these models showed a 22% reduction in the average proportion of variance of the genomic effects across all studied traits for model G + D, and a 1% decrease in the total average variance explained by model G + D (Table S2, Table S3). Similar results have been found in different crops, where the extension of models analogous to G and A with dominance effects (orthogonal or nonorthogonal, assuming or not assuming Hardy–Weinberg equilibrium) has often led to reduced estimates of additive variance components, and sometimes even to a reduction in the total explained variance, falling below the levels achieved by the simpler models G and A ([27]; Costa-Neto, Fritsche-Neto, *et al.*, 2021; [19–21]). While an earlier study showed that dominance variance was overestimated when inbreeding was not taken into account [28], our variance decomposition showed no signs of upwardly biased estimates of dominance variance in model G + D compared to G + D (inb) (Table S2). Our results likely point to potential problems in variance estimation caused by linkage disequilibrium [17, 20], which is prevalent in breeding material such as that contained in the apple REFPOP. Besides the violation of the assumption of linkage equilibrium, the incorrect variance partitioning may have resulted from fitting multiple genetic and genotype-by-environment interaction effects within the framework of multi-environmental genomic prediction, which deserves further investigation. A preliminary analysis outside the scope of this study indicated that orthogonality was restored when conducting analyses on across-location clonal values (results not shown).

Compared to other approaches to modeling nonadditive effects, the NOIA approach retains the advantage of allowing deviations from the Hardy–Weinberg equilibrium [17]. In contrast, the method by VanRaden [4] for constructing standard genomic relationship matrices assumes that the population is unselected and in Hardy–Weinberg equilibrium. However, instead of using allele frequencies from a hypothetical unselected population in Hardy–Weinberg equilibrium, the standard genomic relationship matrix ${\boldsymbol{K}}_{\boldsymbol{G}}$ was computed using observed allele frequencies from our training population. Although this assumption is violated for ${\boldsymbol{K}}_{\boldsymbol{G}}$, our study showed a strong similarity between ${\boldsymbol{K}}_{\boldsymbol{G}}$ and the additive genomic relationship matrix ${\boldsymbol{K}}_{\boldsymbol{A}}$ that was based on the NOIA approach (Fig. 2), along with the near-identical average predictive abilities across traits observed for models G and A (Fig. 5). These outcomes may suggest that any potential violation of Hardy–Weinberg equilibrium in the studied population had minimal impact on genomic prediction. In addition, despite the similarity between ${\boldsymbol{K}}_{\boldsymbol{G}}$ and ${\boldsymbol{K}}_{\boldsymbol{A}}$, differences in the prediction error variance of the genomic-estimated breeding values could arise when using these matrices in genomic prediction models [29]. However, these differences were not investigated in this study.

### Non-genetic effects from envirotyping

As suggested by moderate differences in daily weather variables among years and locations, and the low differentiation between environments within a location in the enviromic relationship matrix, environmental covariates discriminated well between locations but weakly between specific environments. This could likely be explained by the larger number of soil covariates [8] than weather covariates [30] and the lack of variability between years for the soil covariates due to their single measurement at each orchard location in 2016. Additionally, the precipitation variable, which could have aided in distinguishing between environments, had to be excluded from the analysis. This decision was prompted by the confounding of precipitation with irrigation at some apple REFPOP locations. Nevertheless, the enviromic-based effects explained a substantial part of the phenotypic variance, especially for floral emergence known to be strongly affected by the environment [11]. Although a large proportion of phenotypic variance was explained here by the enviromic-based effects, and these effects have been shown to positively influence predictive ability in other crops (Costa-Neto, Fritsche-Neto, *et al.*, 2021; [5]), they have not resulted in any increase in predictive ability for apple traits. For productivity traits such as flowering intensity, which depends on flower bud formation during the previous vegetation season, the models could likely benefit from including prior-year environmental data in the construction of the enviromic matrix.

### Alternative kernels

Similar to previous reports that have shown increased predictive ability when Gaussian kernel and Deep kernel were applied (Costa-Neto, Fritsche-Neto, *et al.*, 2021; [15]), these kernels resulted in a modest but significant improvement in predictive ability of 0.01–0.02 for most of the studied traits. The Gaussian kernel proved particularly suitable for capturing variance attributed to G × E. Model structures based on the Deep kernel generally explained a smaller proportion of phenotypic variance than those using the Gaussian kernel and G-BLUP. This characteristic rendered Deep kernel less suitable for evaluating trait genetic architecture. Nevertheless, the Deep kernel-based models demonstrated improved predictive abilities, equivalent to those of Gaussian kernel-based models. Overall, both alternative kernels proved to be efficient substitutes for G-BLUP.

### Deep learning for genomic prediction

Specifically for each trait and cross-validation fold, the dimensional reduction of the marker dataset to a subset of 1000 SNPs selected by a gradient boosting algorithm, extended with known marker–trait associations, allowed an efficient implementation of a deep learning approach for multi-environmental genomic prediction in apple. The studied deep learning approach combined feature streams derived from marker information with streams incorporating weather and soil variables. It resulted in stream contributions that effectively represented trait genetic architectures described in this and previous studies using statistical genomic prediction models [11].

Our study demonstrated that the applied deep learning approach was particularly well-suited for oligogenic traits. For these traits, governed by a few genes, the dimensionality reduction of the marker dataset allowed important genomic information to be effectively represented. The trait genetic architecture for harvest date was particularly well captured, with a 72% contribution from the SNP stream. Harvest date was previously described as oligogenic trait with significant large-effect marker associations found on chromosomes 3, 10, and 16 using the apple REFPOP dataset [11, 12]. The strongest of these associations on chromosome 3 at 30.7 Mb [11] was located in a major locus *NAC18.1* associated with harvest date and multiple ripening traits [9, 31]. The deep learning genomic prediction model proved efficient in capturing this major locus, as the three SNPs with the highest absolute mean SHAP were located on chromosome 3 at 29.2, 30.7, and 30.8 Mb, the marker AX-115366114 at 30.7 Mb being strongly associated with harvest date according to our previous study [11]. Moreover, the deep learning genomic prediction model outperformed the benchmark statistical genomic prediction model G for harvest date, improving predictive ability by 0.06 and achieving the highest predictive ability among all tested models at 0.75.

Red over color has shown similar predictive ability and trait genetic architecture as harvest date in this and previous studies based on statistical genomic prediction models [11]. The SNPs associated with *MdMYB1* transcription factor on chromosome 9, which regulates red pigmentation of apple skin [32], translated into large absolute mean SHAP values and predictive ability improved by 0.10 compared to model G. Similar results were observed for titratable acidity, where large absolute mean SHAP were found, and the three SNPs with the largest SHAP were located on chromosome 8 at 10.7, 10.8, and 11.8 Mb. Two large-effect loci are known for acidity in apple, namely *Ma* on chromosome 16 and *Ma3* on chromosome 8 [33]. The SNPs on chromosome 8 indicated a strong association with the *Ma3* locus, and they colocalized with the SNP marker predictive for this locus at 10.9 Mb [34]. The maximum relative SHAP contribution for the SNP stream of 80% was reached for titratable acidity. Moreover, the predictive ability of the deep learning genomic prediction model for titratable acidity was improved by 0.07 compared to the statistical genomic prediction model G. Our results for harvest date, red over color, and titratable acidity showed that high relative and absolute SHAP values can serve as predictors of improved deep learning genomic prediction model performance, and that the applied deep learning approach can precisely predict apple traits characterized by oligogenic architecture.

According to Montesinos-López *et al.* [24], the predictive ability of deep learning approaches typically falls below that of conventional models for genomic prediction, unless very large datasets are examined. In our study, the sizes of datasets showed large differences between the three traits, with predictive ability superior to all other compared statistical genomic prediction models (total number of training instances of 12 428 for harvest date, 10 317 for red over color, and 2879 for titratable acidity, Table S1). Although the number of available environments ranged from the minimum of eight for titratable acidity to the maximum of 25 for harvest date, similar improvement in predictive ability was reached for these traits using the applied deep learning approach. As the improvements in predictive ability for harvest date, red over color, and titratable acidity were observed independently from the number of training instances, the size of the phenotypic dataset is unlikely to have affected our predictions. Nevertheless, an additional improvement in predictive ability for the deep learning model may be anticipated by increasing the training population size in terms of the number of genotypes.

### Multi-environmental genomic selection in apple breeding

The establishment of multi-environmental genomic selection in apple has been constrained by several factors, including the costly collection of extensive multi-environmental datasets and computational limitations. The phenotyping efforts in the apple REFPOP yielded an unprecedented dataset in terms of trait–environment combinations [11], which has been expanded in this study with two additional years of phenotyping. This dataset now encompasses phenotypic data for eleven traits across up to 25 environments. The availability of this dataset has enabled the implementation of multi-environmental genomic prediction models within a computationally efficient framework, laying the groundwork for the practical application of multi-environmental genomic selection in apple. Further insights into predictive ability for independent test sets could be gained in the future by assessing the predictive performance on breeding material distinct from the apple REFPOP. Additionally, expanding the training set size may increase predictive ability for some traits [35] and could potentially enable a more accurate estimation of variance components. To expand the dataset by increasing the number of genotypes and environments, new collaborative approaches between breeders are required to generate data capable of overcoming this challenge.

The approach to multi-environmental genomic prediction of apple traits used in this study diverges from the traditional understanding of environments in apple tree cultivation. In practice, apple trees remain stationary in the same location across multiple years. This stationary nature of apple cultivation implies that the effects of yearly climatic variations are superimposed on the same geographical location, whereas the genomic prediction approach treats each year–location combination as a distinct environment. Nevertheless, breeding values for apple genotypes lacking phenotypic information can be predicted across diverse environmental conditions using the genomic prediction models trained in this study.

Among all predictions obtained, the model G + G × E applying Gaussian and Deep kernels improved predictive abilities for most traits (except for titratable acidity and fruit firmness, where it showed results comparable to those of the benchmark model G). Therefore, the model G + G × E proved to be a universally effective solution for multi-environmental genomic prediction in the studied apple traits. Additionally, the G + G × E model, along with other statistical genomic prediction models tested, was outperformed by the applied deep learning approach for three traits with oligogenic genetic architectures (harvest date, titratable acidity, and red over color). Depending on the genetic architecture of the trait, either the G + G × E model or the deep learning approach can be recommended for multi-environmental genomic predictions, leading to informed breeding decisions, and assisting in the selection of cultivars more adaptable to future climates.

## Materials and methods

### Plant material

The apple REFPOP comprised 265 progenies from 27 biparental families generated by European breeding programs, along with 269 diverse accessions [12]. This study focused on five locations: (i) Rillaar, Belgium, (ii) Angers, France, (iii) Laimburg, Italy, (iv) Lleida, Spain, and (v) Waedenswil, Switzerland. At each location, all genotypes were generally represented by two trees and planted in 2016 using a randomized complete block design. Three control genotypes, namely ‘Gala’, ‘Golden Delicious’, and ‘CIVG198’, were replicated up to 22 times at each location. The cultivation followed the common agricultural practices specific to each location, incorporating integrated plant protection methods.

### Phenotyping

Phenotyping of the eleven traits followed the methodology described by Jung *et al.* [11]. Individual trees, representing genotype replicates, were used for trait measurement. Floral emergence was determined in Julian days, marking the date when the first 10% of flowers opened. Flowering intensity was evaluated on a nine-grade scale, indicating the percentage of existing flowers relative to the maximum potential number of flowers. Fruits were harvested on harvest dates, determined in Julian days, based on expert estimates of fruit ripening. Total fruit weight (kg) and fruit number were recorded to assess production per tree. Single fruit weight (g) was estimated by dividing the total fruit weight by the number of fruits. Titratable acidity (g/l), soluble solids content (°Brix), and fruit firmness (g/cm^2^) were measured within one week postharvest using an automated instrument Pimprenelle (Setop, France). Red over color, representing the percentage of red fruit skin, was assessed on a six-grade scale. Russet frequency indicated the percentage of fruits exhibiting russet skin. Further information regarding the evaluation of the eleven traits is available in Jung *et al.* [11]. For the different traits, the assessment spanned a period of up to five years from 2018 to 2022 and was performed at up to five locations.

### Envirotyping

Hourly measurements of temperature (°C) at 2 m above soil level, relative humidity (%), and global radiation (W/m2) were obtained from the weather stations near the apple REFPOP orchards from 2018 to 2022. Precipitation (mm) was not taken into consideration in this study due to irrigation practices in part of the orchard locations.

In each apple REFPOP orchard between May 12 and June 9, 2016, a total of six soil samples were collected from three distinct sampling points and two soil depths (~1–20 cm and 20–40 cm). In the accredited Laboratory for Soil and Plant Analysis of Laimburg Research Centre, Italy, the soil samples were analyzed for (i) organic carbon (% humus); (ii) pH; (iii) carbonate test, expressed as low to medium, high, very high, or no carbonate content; (iv) carbonate requirement (dt/ha CaO); (v) phosphorus (mg/100 g P_2_O_5_); (vi) potassium (mg/100 g K_2_O); (vii) magnesium (mg/100 g); (viii) boron (mg/kg); (ix) manganese (mg/kg); (x) copper (mg/kg); and (xi) zinc (mg/kg).

### Genotyping

As detailed by Jung *et al.* [12], the apple REFPOP underwent genotyping for biallelic SNPs through a dual approach utilizing the Illumina Infinium® 20 K SNP genotyping array [30] and the Affymetrix Axiom® Apple 480 K SNP genotyping array [36]. By employing the Beagle 4.0 software [37] and incorporating pedigree information [38], the obtained SNP sets were integrated through imputation, ultimately yielding a genomic dataset of 303 239 biallelic SNPs. All SNP positions were based on the doubled haploid GDDH13 (v1.1) reference genome [39].

### Phenotypic data preprocessing

Analyses of phenotypic data were conducted to ensure high data quality by addressing low heritability, spatial heterogeneity, and eliminating outliers. The statistical model for the phenotypic data preprocessing was fitted via restricted maximum likelihood using the R package ‘lme4’ (v.1.1–28) [40] as:


(1) \begin{equation*} \boldsymbol{y}=\boldsymbol{X}\boldsymbol{\beta } +\boldsymbol{Zu}+\boldsymbol{\varepsilon} \end{equation*}

where $\boldsymbol{y}$ was the vector of the response variable, $\boldsymbol{X}$ the design matrix for the fixed effects, $\boldsymbol{\beta}$ the vector of the fixed effects, $\boldsymbol{Z}$ was the design matrix for the random effects, $\boldsymbol{u}$ the vector of the random effects assuming $\boldsymbol{u}\sim N\left(0,\boldsymbol{\Sigma} \right)$ with $\boldsymbol{\Sigma}$ being the variance–covariance matrix of the random effects, and $\boldsymbol{\varepsilon}$ the vector of the random errors assuming $\boldsymbol{\varepsilon} \sim N\left(0,{\sigma}_{\varepsilon}^2\boldsymbol{I}\right)$ with ${\sigma}_{\varepsilon}^2$ being the error variance and $\boldsymbol{I}$ the identity matrix.

Separately for each trait and environment (combined factor of location and year), raw phenotypic values for each genotype replicate (total fruit weight and fruit number were log-transformed) were used as response variable to fit a random-effects model with a random effect of genotype following Equation 1. From the variance components of the random-effects model, the environment-specific clonal mean heritability was calculated as:


$$ {H}^2=\frac{\sigma_g^2}{\sigma_g^2+\frac{\sigma_{\varepsilon}^2}{{\overline{n}}_t}} $$

where ${\sigma}_g^2$ was the genotypic variance and ${\overline{n}}_t$ the mean number of genotype replications. The environment-specific clonal mean heritability was used to remove trait–environment combinations with the heritability value <0.1.

To account for spatial variation in the orchards, spatial heterogeneity in the raw phenotypic data was modeled separately for each trait–environment combination using the spatial analysis of field trials with splines (‘SpATS’ (v.1.0–11)) [41] as described by Jung *et al.* [12]. From the fitted SpATS objects, the adjusted phenotypic values of each genotype and the adjusted phenotypic values of each tree were obtained.

The adjusted phenotypic values of each genotype were used as response variable for fitting a mixed-effects model with a fixed effect of environment and a random effect of genotype following Equation 1. Subsequently, the outliers were detected using Bonferroni–Holm test to judge residuals standardized by the rescaled median absolute deviation (BH-MADR) as described by Bernal-Vasquez *et al.* [42]. The identified outliers were removed and the remaining trait- and environment-specific adjusted phenotypic values of each genotype were further denoted as adjusted means. The adjusted means for the eleven studied traits were compared separately for each year and location using the pairwise Pearson’s correlations and significance tests implemented in the R package ‘corrplot’ (v.0.92) [43]. The significance levels of 0.05, 0.01, and 0.001 were Bonferroni-corrected by dividing them by the total number of pairwise comparisons among the eleven traits.

Following Equation 1, the adjusted phenotypic values of each tree served as the response variable in fitting a mixed-effects model, denoted here as the phenotypic model. This model included the fixed effects of environment (E), the random effects of genotype (g), and random effects of genotype-by-environment interaction (g × E). The proportions of phenotypic variance explained by the random effects were extracted from the model fit for comparison with the statistical genomic prediction models.

### Enviromic data preprocessing

The enviromic data were restructured to acquire appropriate inputs for the subsequent modeling. Daily temperature means, daily humidity means, and daily radiation sums were calculated from the hourly measurements. These three daily weather variables were visualized applying local regression curves estimated using Loess with a span of 0.1.

Inspired by Jarquín *et al.* [5], the three daily weather variables were processed to create six environmental covariates by dividing each growing season into two periods based on crop phenology. The two periods were defined separately for each environment. The first period extended for 80 days, concluding on the day when 90% of the genotypes flowered, determined from adjusted means for floral emergence. The second period followed the first until the day when 90% of the genotypes were harvested, as indicated by the adjusted means for harvest date. Different approaches to defining the first period were employed for two environments where adjusted means for floral emergence were unavailable. In the case of the environment ESP.2020, which was excluded due to low heritability, the adjusted phenotypic values of each genotype were used to estimate the day when 90% of the genotypes flowered. For ESP.2022, where floral emergence scores were missing, the end date of the first period was estimated based on varieties cultivated near the apple REFPOP. Daily temperature means, daily humidity means, and daily radiation sums were summed over each respective period, resulting in six environmental covariates. Additionally, 22 environmental covariates were obtained as mean values of eleven soil characteristics calculated per location and the level of soil depth. All 28 environmental covariates were collected in the $q\times z$ matrix of environmental covariates ${\boldsymbol{M}}_{\boldsymbol{W}}$ with $q$ environments and $z$ environmental covariates, which was then scaled and centered to the mean of zero and standard deviation of one.

### Marker matrices

Three marker matrices were constructed based on the genomic dataset of biallelic SNPs. The first matrix followed the standard allele coding, where a SNP was assigned the value 0 when the individual (i.e. genotype) was homozygous for the first allele ($a$), 1 when the genotype was heterozygous, and 2 when the genotype was homozygous for the second allele ($A$). The allele coding can be referred to as coefficients in the marker matrix. Therefore, the $n\times m$ marker matrix of the standard coefficients ${\boldsymbol{M}}_{\boldsymbol{G}}$ with $i=1,\dots, n$ genotypes and $j=1,\dots, m$ markers was:


$$ {\boldsymbol{M}}_{\boldsymbol{G}}=\left[\begin{array}{@{}ccc@{}}{h}_{G_{11}}& \cdots & {h}_{G_{1m}}\\{}\vdots & \ddots & \vdots \\{}{h}_{G_{n1}}& \cdots & {h}_{G_{nm}}\end{array}\right] $$

where the element ${h}_{G_{ij}}$ for the $i$th genotype and $j$th marker was equal to:


$$ {h}_{G_{ij}}=\left\{\begin{array}{@{}c}2\\{}1\\{}0\end{array}\right. \ \ \textrm{for}\ \ \left\{\begin{array}{@{}c} AA\\{} Aa\\{} aa.\end{array}\right. $$

with $AA$, $Aa$, and $aa$ being the combinations of the alleles $a$ and $A$ at the marker $j$. Each column of the matrix ${\boldsymbol{M}}_{\boldsymbol{G}}$ was scaled and centered to the mean of zero and standard deviation of one.

The second and third marker matrices followed the NOIA model [16] as implemented by Vitezica *et al.* [17]. These matrices were estimated from the elements of the marker matrix of the standard coefficients ${\boldsymbol{M}}_{\boldsymbol{G}}$ and had the same dimension. The element ${h}_{A_{ij}}$ for the $n\times m$ marker matrix of additive coefficients ${\boldsymbol{M}}_{\boldsymbol{A}}$ and the element ${h}_{D_{ij}}$ for the $n\times m$ marker matrix of dominance coefficients ${\boldsymbol{M}}_{\boldsymbol{D}}$ were calculated as follows:


$$ {h}_{A_{ij}}=\left\{\begin{array}{@{}c}-\left(-{p}_{Aa}-2{p}_{aa}\right)\\{}-\left(1-{p}_{Aa}-2{p}_{aa}\right)\\{}-\left(2-{p}_{Aa}-2{p}_{aa}\right)\end{array}\right.\ \ \textrm{for}\ \ \left\{\begin{array}{@{}c} AA\\{} Aa\\{} aa\end{array}\right. $$

and


$$ {h}_{D_{ij}}=\left\{\begin{array}{@{}c}-\frac{2{p}_{Aa}{p}_{aa}}{p_{AA}+{p}_{aa}-{\left({p}_{AA}-{p}_{aa}\right)}^2}\\{}\frac{4{p}_{AA}{p}_{aa}}{p_{AA}+{p}_{aa}-{\left({p}_{AA}-{p}_{aa}\right)}^2}\\{}-\frac{2{p}_{AA}{p}_{Aa}}{p_{AA}+{p}_{aa}-{\left({p}_{AA}-{p}_{aa}\right)}^2}\end{array}\right.\\\textrm{for}\ \ \left\{\begin{array}{@{}c} AA\\{} Aa\\{} aa\end{array}\right. $$

with ${p}_{AA}$, ${p}_{Aa}$, and ${p}_{aa}$ being the relative frequencies for the allelic combinations $AA$, $Aa$, and $aa$ at marker $j$.

### Relationship matrices

The marker matrices ${\boldsymbol{M}}_{\boldsymbol{G}}$, ${\boldsymbol{M}}_{\boldsymbol{A}}$, and ${\boldsymbol{M}}_{\boldsymbol{D}}$ and the matrix of environmental covariates ${\boldsymbol{M}}_{\boldsymbol{W}}$ were used to estimate the standard genomic relationship matrix ${\boldsymbol{K}}_{\boldsymbol{G}}$, the additive genomic relationship matrix ${\boldsymbol{K}}_{\boldsymbol{A}}$, the dominance genomic relationship matrix ${\boldsymbol{K}}_{\boldsymbol{D}}$, and the enviromic relationship matrix ${\boldsymbol{K}}_{\boldsymbol{W}}$, respectively. Initially, all relationship matrices were created based on the G-BLUP approach described by VanRaden [4]. The covariance matrix following the G-BLUP approach was obtained as:


$$ \boldsymbol{K}=\frac{{\boldsymbol{M}\boldsymbol{M}}^{\prime }}{tr\left(\boldsymbol{M}{\boldsymbol{M}}^{\prime}\right)/ nrow\left(\boldsymbol{M}\right)} $$

where $\boldsymbol{K}$ was a generic representation of the relationship matrix (${\boldsymbol{K}}_{\boldsymbol{G}}$, ${\boldsymbol{K}}_{\boldsymbol{A}}$, ${\boldsymbol{K}}_{\boldsymbol{D}}$, and ${\boldsymbol{K}}_{\boldsymbol{W}}$); $\boldsymbol{M}$ was a generic representation of the marker matrices ${\boldsymbol{M}}_{\boldsymbol{G}}$, ${\boldsymbol{M}}_{\boldsymbol{A}}$, and ${\boldsymbol{M}}_{\boldsymbol{D}}$ as well as the matrix of environmental covariates ${\boldsymbol{M}}_{\boldsymbol{W}}$; and $nrow$ was the number of genotypes for ${\boldsymbol{M}}_{\boldsymbol{G}}$, ${\boldsymbol{M}}_{\boldsymbol{A}}$, and ${\boldsymbol{M}}_{\boldsymbol{D}}$ or the number of environments for ${\boldsymbol{M}}_{\boldsymbol{W}}$.

Subsequently, two covariance matrix types, namely the Gaussian kernel [44] and Deep kernel [15], were examined as alternatives to the G-BLUP approach. The Gaussian kernel is a nonlinear method based on a bandwidth parameter that controls the decay rate of covariance between genotypes, and the percentile of the square of the Euclidean distance, which is a metric reflecting the genetic distance between genotypes. The Deep kernel is characterized by a nonlinear arc-cosine function, and its covariance matrix is designed to mimic a deep learning model featuring a single hidden layer with many neurons. Applying these alternative approaches, the standard genomic and enviromic relationship matrices based on Gaussian kernel (${\boldsymbol{K}}_{{\boldsymbol{G}}_{\boldsymbol{G}\boldsymbol{K}}}$ and ${\boldsymbol{K}}_{{\boldsymbol{W}}_{\boldsymbol{GK}}}$) and Deep kernel (${\boldsymbol{K}}_{{\boldsymbol{G}}_{\boldsymbol{DK}}}$ and ${\boldsymbol{K}}_{{\boldsymbol{W}}_{\boldsymbol{DK}}}$) were created. The Gaussian kernel and Deep kernel were implemented following the estimation process as detailed by Costa-Neto, Fritsche-Neto, *et al.* (2021).

### Statistical genomic prediction model structures

The relationship matrices were used to create linear mixed model structures for the statistical genomic prediction models. Following Costa-Neto, Fritsche-Neto, *et al.* (2021), the generic model structure was defined as:


(2) \begin{equation*} \boldsymbol{y}=1\boldsymbol{\mu} +{\boldsymbol{X}}_{\boldsymbol{f}}\boldsymbol{\beta} +{\sum}_{\boldsymbol{s}=1}^{\boldsymbol{k}}{\boldsymbol{g}}_{\boldsymbol{s}}+{\sum}_{\boldsymbol{r}=1}^{\boldsymbol{l}}{\boldsymbol{w}}_{\boldsymbol{r}}+\boldsymbol{\varepsilon} \end{equation*}

where $\boldsymbol{y}$ was the vector of the adjusted means for $n$ genotypes across $q$ environments, $1\boldsymbol{\mu}$ was the overall mean, ${\boldsymbol{X}}_{\boldsymbol{f}}$ the design matrix for the fixed effects of environments, $\boldsymbol{\beta}$ the vector of the fixed effects, ${\boldsymbol{g}}_{\boldsymbol{s}}$ the random vector for $s=1,\dots, k$ marker-based effects, ${\boldsymbol{w}}_{\boldsymbol{r}}$ the random vector for $r=1,\dots, l$ enviromic-based effects, and $\boldsymbol{\varepsilon}$ the vector of the random errors assuming $\boldsymbol{\varepsilon} \sim N\left(0,{\sigma}_{\varepsilon}^2\boldsymbol{I}\right)$ with ${\sigma}_{\varepsilon}^2$ being the error variance and $\boldsymbol{I}$ the identity matrix. The effects of environments were modeled as fixed in all model structures tested, consistent with other multi-environmental models that incorporate G × E, as described by, e.g. Lopez-Cruz *et al.* [6] and Costa-Neto, Fritsche-Neto, *et al.* (2021). All model structures were based on the G-BLUP approach to estimating the relationship matrices. When the alternatives to the G-BLUP were used, the model structures were additionally labeled with ‘(GK)’ for the Gaussian kernel and ‘(DK)’ for the Deep kernel. For all three approaches to estimating the relationship matrices, the function get_kernel of the R package ‘EnvRtype’ (v.1.1.1) (Costa-Neto, Galli, *et al.*, 2021) was used to obtain the relationship matrices for genomic prediction.

#### Models G, A, and G + D (random (main) genotypic effects (MM))

Following Equation 2, the model MM accounted for the marker-based effects (${\sum}_{\boldsymbol{s}=1}^{\boldsymbol{k}}{\boldsymbol{g}}_{\boldsymbol{s}}\ne 0$) without applying the enviromic-based effects (${\sum}_{\boldsymbol{r}=1}^{\boldsymbol{l}}{\boldsymbol{w}}_{\boldsymbol{r}}=0$). The ${\boldsymbol{g}}_{\boldsymbol{s}}$ incorporated relationship matrices ${\boldsymbol{K}}_{\boldsymbol{G}}$ (alternatively ${\boldsymbol{K}}_{{\boldsymbol{G}}_{\boldsymbol{G}\boldsymbol{K}}}$ or ${\boldsymbol{K}}_{{\boldsymbol{G}}_{\boldsymbol{DK}}}$), ${\boldsymbol{K}}_{\boldsymbol{A}}$, and ${\boldsymbol{K}}_{\boldsymbol{D}}$ representing random genomic (G), additive (A), and dominance (D) effects, respectively. These effects were applied individually or in combinations, resulting in model structures denoted as G (alternatively G (GK) and G (DK)), A, and G + D.

#### Models G + G × E and G + G × E + D + D × E (single-variance genotype × environment deviation (MDs))

Analogous to the model MM, the model MDs assumed ${\sum}_{\boldsymbol{s}=1}^{\boldsymbol{k}}{\boldsymbol{g}}_{\boldsymbol{s}}\ne 0$ and ${\sum}_{\boldsymbol{r}=1}^{\boldsymbol{l}}{\boldsymbol{w}}_{\boldsymbol{r}}=0$ (Equation 2). In addition to the random effects G and D, the random interaction effects (×) with the vector of environments (E) were included, namely the G × E and D × E. This resulted in model structures G + G × E (alternatively G + G × E (GK) and G + G × E (DK)) and G + G × E + D + D × E.

#### Model G + W (EMM)

The model enviromic-enriched MM (EMM) applied both the marker-based effects $\big({\sum}_{\boldsymbol{s}=1}^{\boldsymbol{k}}{\boldsymbol{g}}_{\boldsymbol{s}}\ne 0\big)$ and the enviromic-based effects (${\sum}_{\boldsymbol{r}=1}^{\boldsymbol{l}}{\boldsymbol{w}}_{\boldsymbol{r}}\ne 0$) (Equation 2). Included were the random effects G and the random enviromic effects (W), the latter being derived through the integration of the relationship matrix ${\boldsymbol{K}}_{\boldsymbol{W}}$ (alternatively ${\boldsymbol{K}}_{{\boldsymbol{W}}_{\boldsymbol{GK}}}$ and ${\boldsymbol{K}}_{{\boldsymbol{W}}_{\boldsymbol{DK}}}$). The resulting model structure was G + W (alternatively G + W (GK) and G + W (DK)).

#### Model G + W + G × W (RNMM)

Building upon the model EMM, the model reaction-norm MM (RNMM) (${\sum}_{\boldsymbol{s}=1}^{\boldsymbol{k}}{\boldsymbol{g}}_{\boldsymbol{s}}\ne 0$ and ${\sum}_{\boldsymbol{r}=1}^{\boldsymbol{l}}{\boldsymbol{w}}_{\boldsymbol{r}}\ne 0$, Equation 2) extended the random enviromic-based effects with a random interaction effect G × W. The obtained model structure was G + W + G × W (alternatively G + W + G × W (GK) and G + W + G × W (DK)).

#### Model G + G × E + W + G × W (RNMDs)

The last of the compared models, the model reaction-norm MDs (RNMDs) (${\sum}_{\boldsymbol{s}=1}^{\boldsymbol{k}}{\boldsymbol{g}}_{\boldsymbol{s}}\ne 0$ and ${\sum}_{\boldsymbol{r}=1}^{\boldsymbol{l}}{\boldsymbol{w}}_{\boldsymbol{r}}\ne 0$, Equation 2), combined the random marker-based effects G and G × E with the random enviromic-based effects W and G × W in a single model structure G + G × E + W + G × W (alternatively G + G × E + W + G × W (GK) and G + G × E + W + G × W (DK)).

#### Fixed effect of inbreeding

The design matrix for the fixed effects ${\boldsymbol{X}}_{\boldsymbol{f}}$ (Equation 2) was based on the vector of environments (E) for all model structures tested in this study. As described by previous authors [20, 28], including an inbreeding coefficient as fixed effect can account for directional dominance effects and help to avoid overestimating the proportion of variance explained by the dominance model components. Hence, the model MM was additionally extended with the fixed effect of inbreeding contained in parameter ${\boldsymbol{X}}_{\boldsymbol{f}}$, which was incorporated in the model structures denoted as G (inb) and G + D (inb). The inbreeding coefficient for each genotype was estimated from the marker matrix ${\boldsymbol{M}}_{\boldsymbol{G}}$, calculated as the relative frequency of the homozygous allelic combinations $AA$ and $aa$ across all markers.

### Deep learning approach

The deep learning genomic prediction model was designed to be able to receive both genotypic and environmental data in the form of four streams. Genotypic data underwent feature selection in two different ways, generating input data for two different streams of the model: SNP stream and PC stream. First, to represent specific genetic variation, the most relevant 1000 SNPs for each trait and fold were extracted from the marker matrix ${\boldsymbol{M}}_{\boldsymbol{G}}$ with a gradient-boosting regressor. The response variable for the gradient-boosting model was derived from the means of the random effects of genotypes, which were extracted from a mixed-effects model. This mixed-effects model followed Equation 1, incorporating fixed effects of the environment (E) and random effects of genotype (g). Additionally, the SNPs associated with the studied traits as reported by Jung *et al.* [11] were added to the existing pool of 1000 SNPs within the SNP stream. Second, using the principal component analysis in related samples (PC-AiR) method [25], 58 PCs capturing 100% of the genetic variation were extracted and used as input to represent the overall genetic variation. Daily weather variables and soil environmental covariates directly constituted the input for the weather and soil streams, respectively. The adjusted phenotypic means served as the response variables. All stream and response variables were scaled between −1 and 1. The model architecture was designed using ‘TensorFlow’ (v.2.10.0) and ‘Keras’ (v.2.10.0). All streams consisted of a variable number of dense layers except for the weather stream. In this case, the first layers were long short-term memory (LSTM), which excel at processing sequential data. The four streams processed the data independently and were concatenated after several layers. Further dense layers were placed before the output neuron to allow for data integration. For specific details on the model architecture, please refer to the provided GitHub link (https://github.com/MichaelaJung/Integrative-prediction). Models for each trait were trained and evaluated at different learning rates (1e^−4^, 1e^−5^, and 5e^−6^). When the training loss stopped improving, the training was stopped. The appropriate learning rate was decided for each trait based on the highest correlation and the lowest root mean squared error.

### Genomic prediction

All statistical and deep learning genomic prediction models were iteratively fitted in a 5-fold cross-validation that was repeated five times, with genotypes being allocated to folds randomly and without replacement, resulting in 25 runs of each tested model. All models were applied using the same genotype allocations for each fold. The statistical genomic prediction model structures were solved using Bayesian hierarchical modeling implemented in the R package ‘BGGE’ (v.0.6.5) [13]. The statistical genomic prediction models underwent 10 000 iterations of the Gibbs sampler, employing a thinning of 3 and discarding the initial 1000 samples as burn-in.

### Relative contribution of model components

For the statistical genomic prediction models, each model fit from the cross-validation was used to obtain the proportions of variance explained by the various random effects. To explain the deep learning model predictions with respect to each input feature (e.g. a SNP or weather variable), the ‘GradientExplainer’ function from the ‘shap’ package (v.0.42.1) [45] was used to calculate approximated SHAP. It uses the gradients of the model to approximate SHAP values for each feature, which estimates their contribution to the prediction. SHAP values were calculated for every instance of every test fold in each repetition of the cross-validation. The absolute values of each feature were averaged to obtain absolute mean SHAP values per feature. Furthermore, to investigate the contribution of each stream to the prediction, the absolute mean SHAP values were summed for every fold, and the relative SHAP contribution of each stream was obtained as a percentage.

### Assessment of predictive ability

For every statistical and deep learning genomic prediction model and trait, 25 estimates of predictive ability were generated for each environment, calculated as Pearson’s correlation coefficient between the adjusted means and predicted values. This resulted in 200 predictive ability estimates for titratable acidity, soluble solids content, and fruit firmness, 500 for russet frequency, 525 for red over color, 575 for floral emergence and flowering intensity, and 625 for harvest date, total fruit weight, number of fruits, and single fruit weight. Average predictive ability across traits was calculated by averaging all estimates of predictive ability for each model.

Four models were selected for an in-depth comparison based on their performance and characteristics. The selection criteria included improvements of at least 0.01 in average predictive ability across traits compared to the benchmark model G. Additionally, statistical genomic prediction models that explained a large proportion of variance were prioritized, with a preference for simpler model structures over more complex ones. For each of these models, both the average predictive ability and the distribution of predictive abilities were visualized and compared with those of the model G for every trait.

All statistical analyses in this work were implemented in R (v.4.1.3) [46]. The code for implementing, training, using, and explaining the deep learning genomic prediction models was written in Python (v.3.9.16).

## Supplementary Material

Web_Material_uhae319

## Data Availability

All SNP genotypic data used in this study have been deposited in the INRAe dataset archive at https://doi.org/10.15454/IOPGYF and https://doi.org/10.15454/1ERHGX. The raw phenotypic data are available in the INRAe dataset archive at https://doi.org/10.15454/VARJYJ. The code underlying this article is available in GitHub at https://github.com/MichaelaJung/Integrative-prediction. The phenotypic, enviromic, and imputed genomic data formatted as input files for the provided code are available in Zenodo at 10.5281/zenodo.14191209.

## References

[1] Meuwissen THE, Hayes BJ, Goddard ME. Prediction of total genetic value using genome-wide dense marker maps. Genetics. 2001;157:1819–29 11290733 10.1093/genetics/157.4.1819 PMC1461589

[2] García-Ruiz A, Cole JB, VanRaden PM. et al. Changes in genetic selection differentials and generation intervals in US Holstein dairy cattle as a result of genomic selection. Proc Natl Acad Sci. 2016;113:E3995–4004 27354521 10.1073/pnas.1519061113 PMC4948329

[3] Voss-Fels KP, Cooper M, Hayes BJ. Accelerating crop genetic gains with genomic selection. Theor Appl Genet. 2019;132:669–86 30569365 10.1007/s00122-018-3270-8

[4] VanRaden PM . Efficient methods to compute genomic predictions. J Dairy Sci. 2008;91:4414–23 18946147 10.3168/jds.2007-0980

[5] Jarquín D, Crossa J, Lacaze X. et al. A reaction norm model for genomic selection using high-dimensional genomic and environmental data. Theor Appl Genet. 2014;127:595–607 24337101 10.1007/s00122-013-2243-1 PMC3931944

[6] Lopez-Cruz M, Crossa J, Bonnett D. et al. Increased prediction accuracy in wheat breeding trials using a marker × environment interaction genomic selection model. G3 (Bethesda). 2015;5:569–82 25660166 10.1534/g3.114.016097 PMC4390573

[7] Kostick SA, Bernardo R, Luby JJ. Genomewide selection for fruit quality traits in apple: breeding insights gained from prediction and postdiction. Hortic Res. 2023;10:uhad088 37334180 10.1093/hr/uhad088 PMC10273070

[8] Kumar S, Chagné D, Bink MCAM. et al. Genomic selection for fruit quality traits in apple (*Malus × domestica* Borkh.). PLoS One. 2012;7:e36674 22574211 10.1371/journal.pone.0036674 PMC3344927

[9] Migicovsky Z, Gardner KM, Money D. et al. Genome to phenome mapping in apple using historical data. Plant Genome. 2016;9:10.3835/plantgenome2015.11.0113 27898813

[10] Muranty H, Troggio M, Sadok IB. et al. Accuracy and responses of genomic selection on key traits in apple breeding. Hortic Res. 2015;2:15060 26744627 10.1038/hortres.2015.60 PMC4688998

[11] Jung M, Keller B, Roth M. et al. Genetic architecture and genomic predictive ability of apple quantitative traits across environments. Hortic Res. 2022;9:uhac028 35184165 10.1093/hr/uhac028 PMC8976694

[12] Jung M, Roth M, Aranzana MJ. et al. The apple REFPOP—a reference population for genomics-assisted breeding in apple. Hortic Res. 2020;7:189 33328447 10.1038/s41438-020-00408-8 PMC7603508

[13] Granato I, Cuevas J, Luna-Vázquez F. et al. BGGE: a new package for genomic-enabled prediction incorporating genotype × environment interaction models. G3 (Bethesda). 2018;8:3039–47 30049744 10.1534/g3.118.200435 PMC6118304

[14] Pérez P, de los Campos G. Genome-wide regression and prediction with the BGLR statistical package. Genetics. 2014;198:483–95 25009151 10.1534/genetics.114.164442 PMC4196607

[15] Cuevas J, Montesinos-López O, Juliana P. et al. Deep kernel for genomic and near infrared predictions in multi-environment breeding trials. G3 (Bethesda). 2019;9:2913–24 31289023 10.1534/g3.119.400493 PMC6723142

[16] Álvarez-Castro JM, Carlborg Ö. A unified model for functional and statistical epistasis and its application in quantitative trait loci analysis. Genetics. 2007;176:1151–67 17409082 10.1534/genetics.106.067348 PMC1894581

[17] Vitezica ZG, Legarra A, Toro MA. et al. Orthogonal estimates of variances for additive, dominance, and epistatic effects in populations. Genetics. 2017;206:1297–307 28522540 10.1534/genetics.116.199406 PMC5500131

[18] Vitezica ZG, Varona L, Legarra A. On the additive and dominant variance and covariance of individuals within the genomic selection scope. Genetics. 2013;195:1223–30 24121775 10.1534/genetics.113.155176 PMC3832268

[19] Kumar S, Molloy C, Muñoz P. et al. Genome-enabled estimates of additive and nonadditive genetic variances and prediction of apple phenotypes across environments. G3 (Bethesda). 2015;5:2711–8 26497141 10.1534/g3.115.021105 PMC4683643

[20] Roth M, Beugnot A, Mary-Huard T. et al. Improving genomic predictions with inbreeding and nonadditive effects in two admixed maize hybrid populations in single and multienvironment contexts. Genetics. 2022;220:iyac018 35150258 10.1093/genetics/iyac018 PMC8982028

[21] Yadav S, Wei X, Joyce P. et al. Improved genomic prediction of clonal performance in sugarcane by exploiting non-additive genetic effects. Theor Appl Genet. 2021;134:2235–52 33903985 10.1007/s00122-021-03822-1 PMC8263546

[22] Cooper M, Messina CD, Podlich D. et al. Predicting the future of plant breeding: complementing empirical evaluation with genetic prediction. Crop Pasture Sci. 2014;65:311–36

[23] Resende RT, Piepho H-P, Rosa GJM. et al. Enviromics in breeding: applications and perspectives on envirotypic-assisted selection. Theor Appl Genet. 2021;134:95–112 32964262 10.1007/s00122-020-03684-z

[24] Montesinos-López OA, Montesinos-López A, Pérez-Rodríguez P. et al. A review of deep learning applications for genomic selection. BMC Genomics. 2021;22:19 33407114 10.1186/s12864-020-07319-x PMC7789712

[25] Kick DR, Wallace JG, Schnable JC. et al. Yield prediction through integration of genetic, environment, and management data through deep learning. G3 (Bethesda). 2023;13:jkad006 36625555 10.1093/g3journal/jkad006 PMC10085787

[26] Jurado-Ruiz F, Rousseau D, Botía JA. et al. GenoDrawing: an autoencoder framework for image prediction from SNP markers. Plant Phenomics. 2023;5:0113 38239740 10.34133/plantphenomics.0113 PMC10795539

[27] Amadeu RR, Ferrão LFV, Oliveira I. et al. Impact of dominance effects on autotetraploid genomic prediction. Crop Sci. 2020;60:656–65

[28] Vitezica ZG, Reverter A, Herring W. et al. Dominance and epistatic genetic variances for litter size in pigs using genomic models. Genet Sel Evol. 2018;50:71 30577727 10.1186/s12711-018-0437-3 PMC6303964

[29] Strandén I, Christensen OF. Allele coding in genomic evaluation. Genet Sel Evol. 2011;43:25 21703021 10.1186/1297-9686-43-25 PMC3154140

[30] Bianco L, Cestaro A, Sargent DJ. et al. Development and validation of a 20K single nucleotide polymorphism (SNP) whole genome genotyping array for apple (*Malus × domestica* Borkh). PLoS One. 2014;9:e110377 25303088 10.1371/journal.pone.0110377 PMC4193858

[31] Watts S, Migicovsky Z, Myles S. Large-scale apple GWAS reveals NAC18.1 as a master regulator of ripening traits. Fruit Res. 2023;3:32

[32] Takos AM, Jaffé FW, Jacob SR. et al. Light-induced expression of a MYB gene regulates anthocyanin biosynthesis in red apples. Plant Physiol. 2006;142:1216–32 17012405 10.1104/pp.106.088104 PMC1630764

[33] Verma S, Evans K, Guan Y. et al. Two large-effect QTLs, Ma and Ma3, determine genetic potential for acidity in apple fruit: breeding insights from a multi-family study. Tree Genet Genomes. 2019;15:18

[34] Rymenants M, van de Weg E, Auwerkerken A. et al. Detection of QTL for apple fruit acidity and sweetness using sensorial evaluation in multiple pedigreed full-sib families. Tree Genet Genomes. 2020;16:71

[35] Minamikawa MF, Kunihisa M, Moriya S. et al. Genomic prediction and genome-wide association study using combined genotypic data from different genotyping systems: application to apple fruit quality traits. Hortic Res. 2024;11:uhae131 38979105 10.1093/hr/uhae131 PMC11228094

[36] Bianco L, Cestaro A, Linsmith G. et al. Development and validation of the Axiom®Apple480K SNP genotyping array. Plant J. 2016;86:62–74 26919684 10.1111/tpj.13145

[37] Browning SR, Browning BL. Rapid and accurate haplotype phasing and missing-data inference for whole-genome association studies by use of localized haplotype clustering. Am J Hum Genet. 2007;81:1084–97 17924348 10.1086/521987 PMC2265661

[38] Muranty H, Denancé C, Feugey L. et al. Using whole-genome SNP data to reconstruct a large multi-generation pedigree in apple germplasm. BMC Plant Biol. 2020;20:2 31898487 10.1186/s12870-019-2171-6 PMC6941274

[39] Daccord N, Celton J-M, Linsmith G. et al. High-quality de novo assembly of the apple genome and methylome dynamics of early fruit development. Nat Genet. 2017;49:1099–106 28581499 10.1038/ng.3886

[40] Bates D, Mächler M, Bolker B. et al. Fitting linear mixed-effects models using lme4. J Stat Softw. 2015;67:1–48

[41] Rodríguez-Álvarez MX, Boer MP, van Eeuwijk FA. et al. Correcting for spatial heterogeneity in plant breeding experiments with P-splines. Spat Stat. 2018;23:52–71

[42] Bernal-Vasquez A-M, Utz HF, Piepho H-P. Outlier detection methods for generalized lattices: a case study on the transition from ANOVA to REML. Theor Appl Genet. 2016;129:787–804 26883044 10.1007/s00122-016-2666-6

[43] Wei T, Simko V. R package “corrplot”: visualization of a correlation matrix. 2021. https://github.com/taiyun/corrplot

[44] González-Camacho JM, de los Campos G, Pérez P. et al. Genome-enabled prediction of genetic values using radial basis function neural networks. Theor Appl Genet. 2012;125:759–71 22566067 10.1007/s00122-012-1868-9 PMC3405257

[45] Lundberg SM, Lee S-I. A unified approach to interpreting model predictions. In: Guyon I, Luxburg UV, Bengio S, Wallach H, Fergus R, Vishwanathan S, Garnett R, eds. Advances in Neural Information Processing Systems (Vol. 30). Curran Associates, Inc., 2017,

[46] R Core Team . (2022). R: A Language and Environment for Statistical Computing. R Foundation for Statistical Computing. http://www.R-project.org/

[47] Costa-Neto G, Fritsche-Neto R, Crossa J. Nonlinear kernels, dominance, and envirotyping data increase the accuracy of genome-based prediction in multi-environment trials. Heredity. 2021a;126:92–106 32855544 10.1038/s41437-020-00353-1 PMC7852533

[48] Costa-Neto G, Galli G, Carvalho HF. et al. EnvRtype: a software to interplay enviromics and quantitative genomics in agriculture. G3 (Bethesda). 2021b;11:jkab040 33835165 10.1093/g3journal/jkab040 PMC8049414

