# Supplementary material for: Integrative multi-environmental genomic prediction in apple
Source: Hortic Res. 2024 Nov 20;12(2):uhae319. doi: 10.1093/hr/uhae319 (PMC11879405; doi:10.1093/hr/uhae319)
Supplement: Web_Material_uhae319 [file web_material_uhae319.zip › 20240904_Supplemental_figures.pdf]

Integrative multi-environmental genomic prediction in apple

Michaela Jung, Carles Quesada-Traver, Morgane Roth, Maria José Aranzana, Hélène Muranty, Marijn Rymenants, Walter Guerra, Elias Holzknecht, Nicole Pradas, Lidia Lozano, Frédérique Didelot, François Laurens, Steven Yates, Bruno Studer, Giovanni AL Broggini, Andrea Patocchi

Supplemental figures

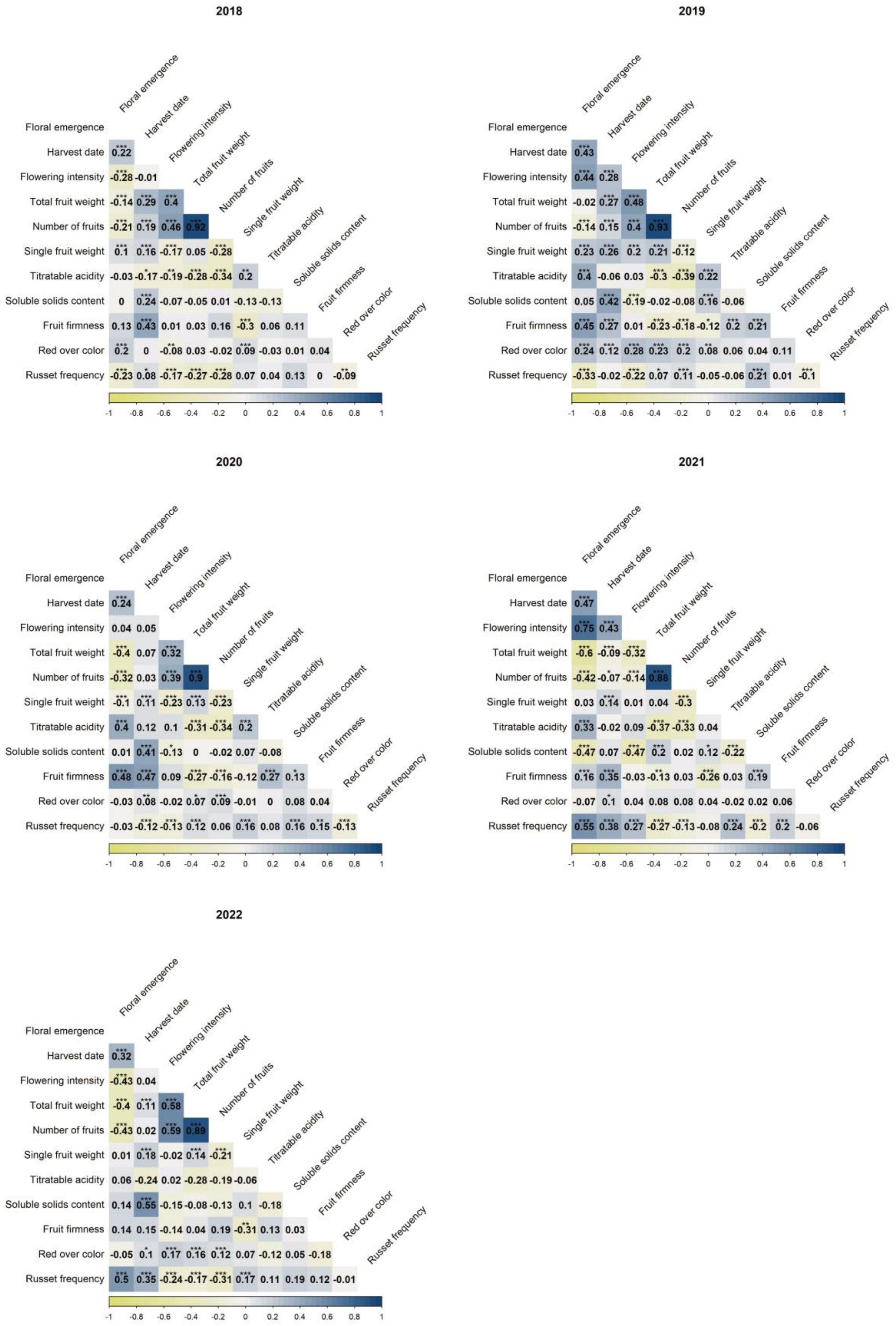

Figure S1: Pairwise Pearson's correlations of the adjusted means for eleven traits in five years (2018–2022) across locations. The asterisks indicate significant correlations at Bonferroni-corrected significance levels: * (α = 0.05), ** (α = 0.01), and *** (α = 0.001).

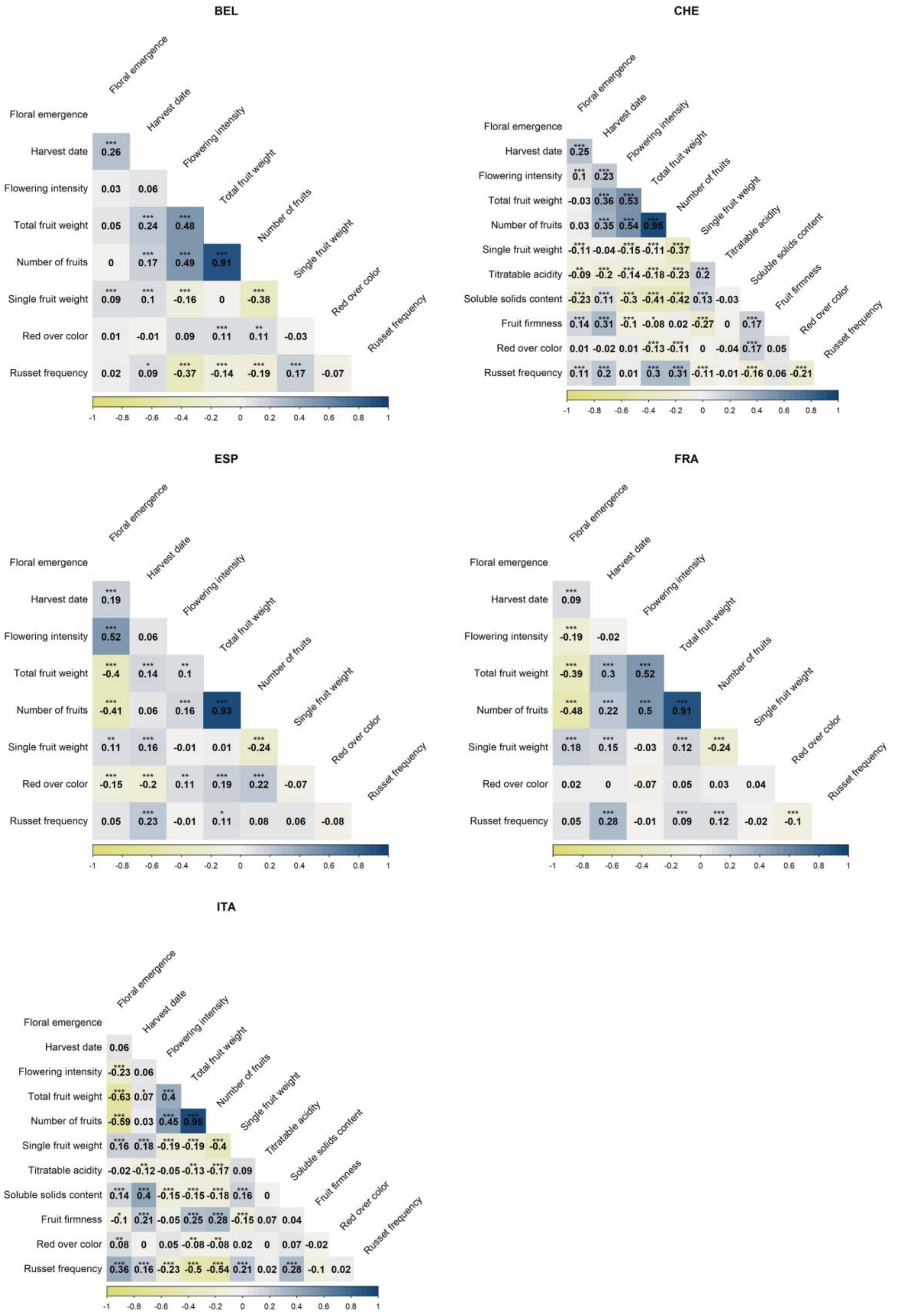

Figure S2: Pairwise Pearson's correlations of the adjusted means for eleven traits and five locations across years. The asterisks indicate significant correlations at Bonferroni-corrected significance levels: * (α = 0.05), ** (α = 0.01), and *** (α = 0.001). The locations correspond to Belgium (BEL), Switzerland (CHE), Spain (ESP), France (FRA) and Italy (ITA).

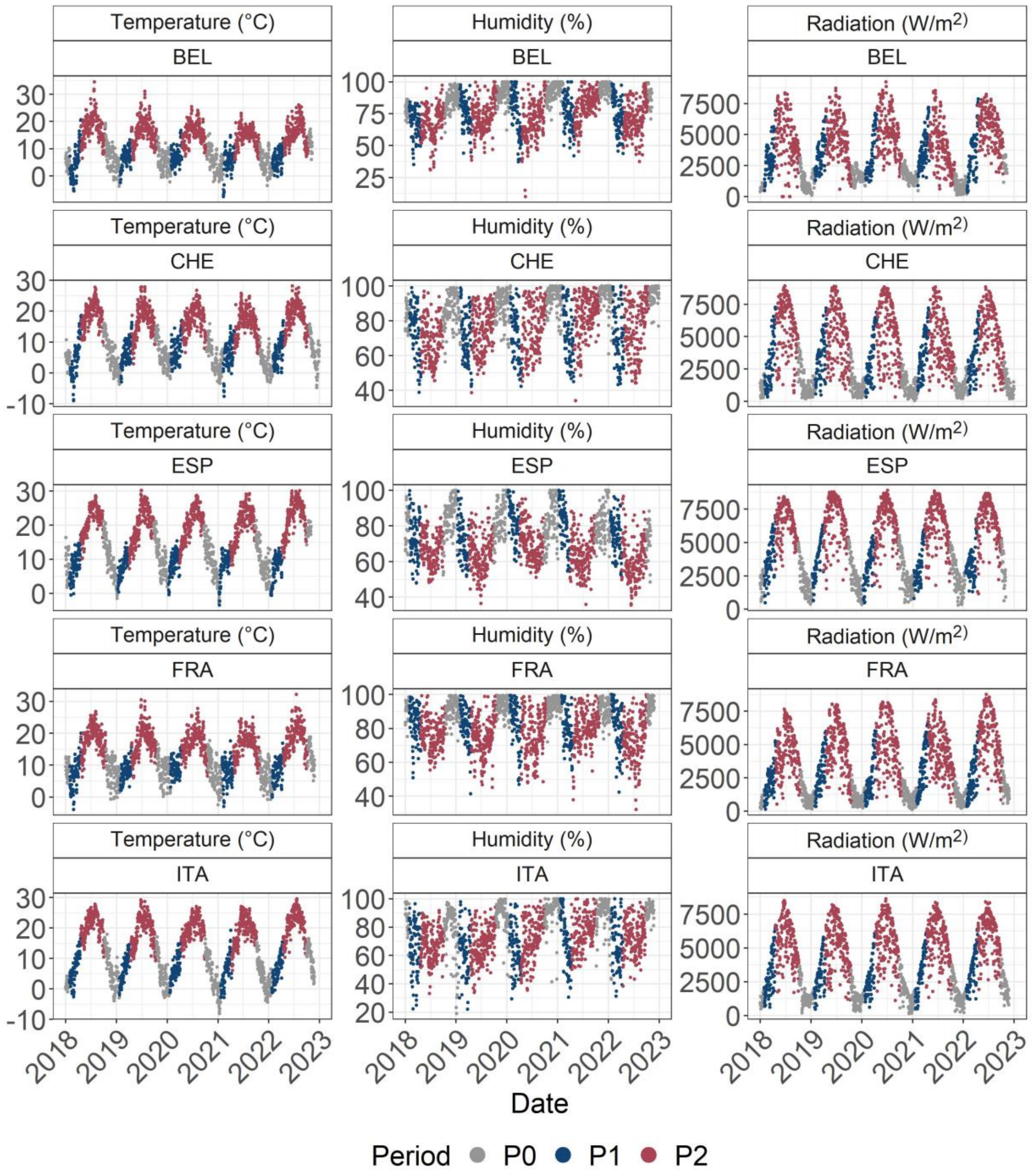

Figure S3: Daily temperature means, daily humidity means, and daily radiation sums for five locations and five years included in the study. The daily values are colored based on periods estimated from crop phenology. The first period (P1) extended for 80 days, concluding on the day when 90% of the genotypes flowered, determined from adjusted means for floral emergence. The second period (P2) followed the first until the day when 90% of the genotypes were harvested, as indicated by the adjusted means for harvest date. Daily values for P0 were excluded from the modelling process. The locations correspond to Belgium (BEL), Switzerland (CHE), Spain (ESP), France (FRA) and Italy (ITA).

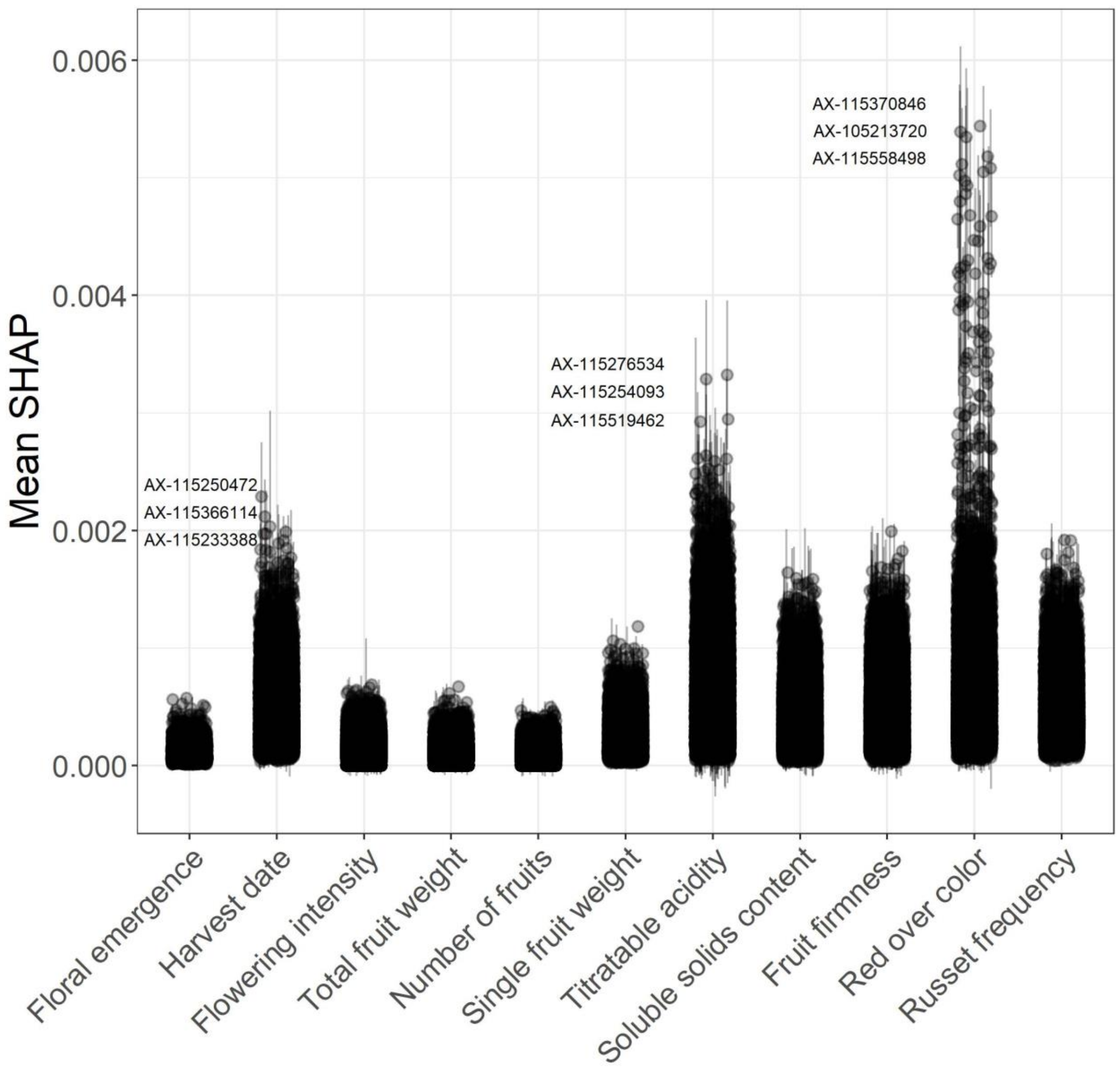

Figure S4: Absolute mean Shapley additive explanations (SHAP) for the SNP stream of the deep learning model. The labels (SNP numbers prefixed with AX) show names of the three most important SNPs for three traits with the largest absolute mean SHAP of the SNP stream. The error bars represent the standard deviation around the mean.

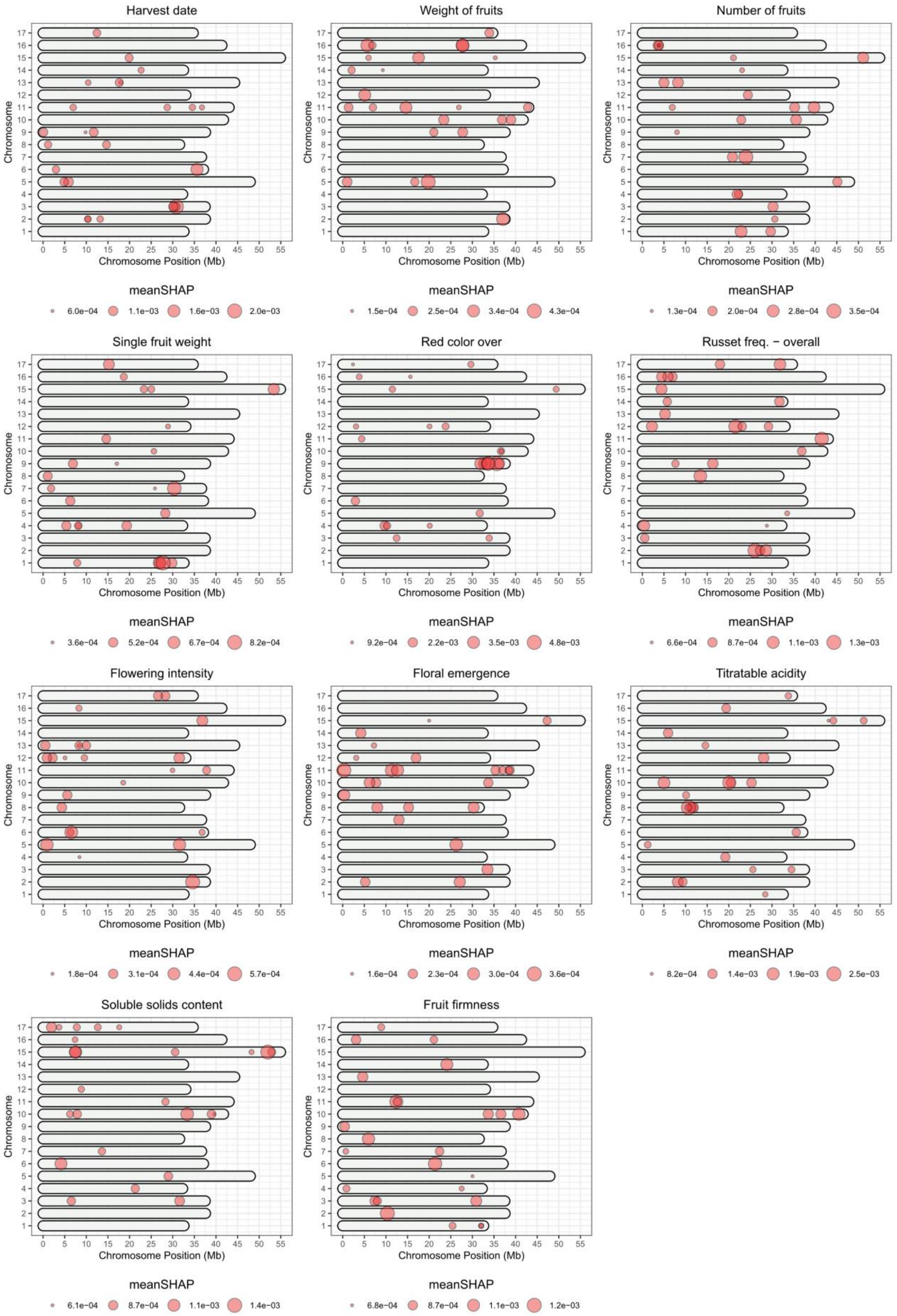

Figure S5: Chromosome positions for 25 SNPs with the highest absolute mean Shapley additive explanations (SHAP) found for each of the eleven studied traits.
